# GROWTH-REGULATING FACTOR 9 negatively regulates arabidopsis leaf growth by controlling *ORG3* and restricting cell proliferation in leaf primordia

**DOI:** 10.1371/journal.pgen.1007484

**Published:** 2018-07-09

**Authors:** Mohammad Amin Omidbakhshfard, Ushio Fujikura, Justyna Jadwiga Olas, Gang-Ping Xue, Salma Balazadeh, Bernd Mueller-Roeber

**Affiliations:** 1 University of Potsdam, Institute of Biochemistry and Biology, Potsdam‐Golm, Germany; 2 CSIRO Plant Industry, St. Lucia, Australia; 3 Max‐Planck Institute of Molecular Plant Physiology, Potsdam‐Golm, Germany; 4 Center of Plant Systems Biology and Biotechnology, Department Plant Development, Plovdiv, Bulgaria; Univerisity of Pennsylvania, UNITED STATES

## Abstract

Leaf growth is a complex process that involves the action of diverse transcription factors (TFs) and their downstream gene regulatory networks. In this study, we focus on the functional characterization of the *Arabidopsis thaliana* TF GROWTH-REGULATING FACTOR9 (GRF9) and demonstrate that it exerts its negative effect on leaf growth by activating expression of the bZIP TF *OBP3-RESPONSIVE GENE 3* (*ORG3*). While *grf9* knockout mutants produce bigger incipient leaf primordia at the shoot apex, rosette leaves and petals than the wild type, the sizes of those organs are reduced in plants overexpressing *GRF9* (*GRF9ox*). Cell measurements demonstrate that changes in leaf size result from alterations in cell numbers rather than cell sizes. Kinematic analysis and 5-ethynyl-2'-deoxyuridine (EdU) incorporation assay revealed that *GRF9* restricts cell proliferation in the early developing leaf. Performing *in vitro* binding site selection, we identified the 6-base motif 5'-CTGACA-3' as the core binding site of GRF9. By global transcriptome profiling, electrophoretic mobility shift assay (EMSA) and chromatin immunoprecipitation (ChIP) we identified *ORG3* as a direct downstream, and positively regulated target of GRF9. Genetic analysis of *grf9 org3* and *GRF9ox org3* double mutants reveals that both transcription factors act in a regulatory cascade to control the final leaf dimensions by restricting cell number in the developing leaf.

## Author summary

Leaves are central plant organs that determine photosynthetic efficiency and thereby biomass production, yet the molecular mechanisms driving and controlling their growth and development are still far from being completely understood. Leaf growth—like other biological processes in different organisms—is controlled by an extensive network of genes that encode proteins of diverse cellular functions. Proteins controlling the activity of genes, called transcription factors, are particularly important for staging developmental processes. Here, we report how GRF9, a member of the so-called Growth-Regulating Factor (GRF) family of transcriptions factors, controls growth of leaves in the widely-studied model plant *Arabidopsis thaliana*, commonly known as thale cress. We show that GRF9 negatively controls final leaf dimensions by restricting cell number in the leaf primordium, while the size of the leaf cells remains unaltered. Using molecular techniques, we discovered that GRF9 acts within a regulatory cascade with another transcription factor, called ORG3. Furthermore, we show that GRF9 and ORG3 negatively control the organ size of flower petals, demonstrating that both transcription factors have a more general role in regulating organ size. Our study is one of the first reports to demonstrate a negative role of GRF transcription factors for organ growth.

## Introduction

Leaves are central photosynthetic organs of terrestrial plants; they determine photosynthesis efficiency and biomass production [1]. The development of leaves is complex and involves the action of many different regulatory proteins including transcription factors [2–4]. A three-phase model has been proposed by which the cells at the shoot apical meristem (SAM) develop into a mature leaf, involving the initiation of a primordium derived from leaf founder cells, primary morphogenesis (cell proliferation), and secondary morphogenesis (elemental expansion); all three phases ultimately affect leaf size [4–10]. In *Arabidopsis thaliana*, phase one starts by the initiation of leaf primordia from cells located within the peripheral zone of the SAM, a process that lasts 2–3 days [3,11]. The transition from a leaf primordium with about 100 cells to a leaf with several thousand cells occurs during primary morphogenesis whereby massive cell proliferation (cell growth and division) occurs. In Arabidopsis leaves, the cell proliferation phase typically lasts 7–9 days. The rate and duration of cell proliferation are two important parameters that strongly affect final leaf size and shape [3,11]. Eventually, cell proliferation ceases in a basipetal (leaf tip to base) manner and secondary morphogenesis starts. Once initiated, the transition between cell proliferation and elemental expansion occurs rather abruptly, within a few days; the zone were cell proliferation ceases and cell elongation starts demarcates the so-called cell cycle arrest front (AF) [11–14]. AF progression is another major controlling step in determining leaf morphogenesis and final leaf size [4,12,15–17]. Secondary morphogenesis represents the longest phase of leaf development; it continues until the leaf reaches its final size. In this phase cells only expand [3,5,14].

Leaf growth and development is controlled by an extensive network of genes encoding different types of regulatory proteins, including many transcription factors (TFs) and several microRNAs [3,4,6,9,14,17–20]. Among them, GROWTH-REGULATING FACTORs (GRFs) represent a plant-specific TF family which has nine members in *Arabidopsis thaliana* (GRF1 –GRF9; [21]). Most members of this TF family are expressed in growing tissues, including leaves [21–24]. Functional and molecular analyses have shown that several GRFs contribute to the regulation of cell proliferation in leaf primordia and to organ separation in the shoot apical meristem [16,17,21–28]. GRFs do so by forming protein complexes with GRF-interacting factors (GIFs), which are transcriptional co-activators that function in determining leaf sizes [17,22,29–34]. The function of GRFs is further controlled by *microRNA396* (*miR396*) which targets a number of GRFs including *GRF1*, *2*, *3*, *4*, *7*, *8*, and *9* to control their expression [16,24,35–38].

Additionally, GRFs have been reported to coordinate plant growth with stress responses [38,39], to affect root and flower development [27,37,38,40,41], and to control plant longevity [24,42]. Interestingly, in contrast to the known positive functions of GRFs in cell proliferation, a recent study in maize (*Zea mays*) showed that *ZmGRF10* functions as a negative regulator of cell proliferation in leaves [34]. The authors showed that overexpression of *ZmGRF10* results in smaller leaves due to a fewer number of cells which may result from a dominant negative effect of this protein on leaf growth by assembling an inactive GRF-GIF complex [17,34].

The establishment of the cell cycle arrest front (AF) is a complex cellular process that is not understood in its details so far. A known regulator of the process is the TCP transcription factor CINCINNATA (CIN), which controls the progression of the mitotic arrest front in snapdragon [43]. Furthermore, cycling cells were closer to the base of developing leaves in *miR396b* overexpressors (*35S*:*miR396b*) than in the wild type, suggesting that this microRNA contributes to controlling the position of the AF, possibly by inhibiting *GRF* transcripts [16]. In accordance with this, *miR396* expression shows a gradient along the leaf axis, with a higher expression at the distal leaf part than the base; during leaf growth, the area of high *miR396* expression extends towards the leaf base thereby progressively inhibiting *GRF* expression in the more proximal organ parts [16].

The importance of GRFs for setting the AF is further substantiated by the finding that the expression and protein levels of all three GIFs (GIF1—GIF3) in Arabidopsis are well correlated with changes in the cell cycle arrest front [22,31,44]. In accordance with this, overexpression of *GIF1* (also known as *AN3*, *ANGUSTIFOLIA3*) delays the progression of the AF towards the base of the leaf [33]. Similarly, overexpression of *miR396*-resistant *GRF1* in maize results in an increased basal division zone in leaves and an increase of leaf length [45].

A further known player involved in establishing the AF during leaf development in Arabidopsis is KLUH/CYP78A5 (KLU), which appears to be involved in the formation of a mobile growth factor (MGF) and generating a concentration gradient of MGF in leaves [15]. The proposed mobile factor has not been identified so far, leaving open the question how KLU controls the setting of the AF.

Here, we report that *GRF9* from *Arabidopsis thaliana* functions as a negative regulator of leaf growth by restricting cell number within the incipient leaf primordium and restraining cell proliferation in the developing leaf. We also found direct binding of GRF9 to the promoter of *OBP3-RESPONSIVE GENE 3* (*ORG3*), which has previously been shown to play a role in the early stages of leaf development [7], establishing a previously unknown GRF9 –*ORG3* regulatory cascade.

## Results

### Transcriptional pattern of the *GRF9* gene

As *GRF9* is not represented on the Arabidopsis ATH1 microarray, information about its expression pattern is limited. We therefore fused the *GRF9* promoter (~1.5-kb promoter upstream the translation initiation codon) to the β*-GLUCURONIDASE* (*GUS*) reporter gene and tested the transcriptional activity of *GRF9* in transgenic Arabidopsis plants (hereafter, *Pro*_*GRF9*_:*GUS*). We observed *GRF9* promoter-driven reporter activity throughout the entire young developing leaf of 3- to 5-day-old seedlings, and in the vascular tissue of cotyledons (**S1A and S1B Fig**). A more detailed analysis of 7- to 9-day-old seedlings revealed that *GRF9* transcriptional activity is mostly restricted to the basal part of the developing leaf in which cells are still actively dividing (**S1C Fig**). In 10- to 12-day-old *Pro*_*GRF9*_:*GUS* seedlings, reporter activity decreases in the leaf blade and remains mostly limited to the vascular tissues (**S1D Fig**). Similarly, reporter activity in mature leaves is mostly restricted to vascular strands (**S1E and S1F Fig**). Moreover, *GRF9* transcriptional activity is dominant in the expanding zone of the roots and low, if detectable, in root tips (**S1G and S1H Fig**). In addition to vegetative tissues, *GRF9* promoter-driven reporter activity is evident in reproductive parts of the plants, i.e. flowers (mostly carpels) and the abscission zone of siliques (**S1I to S1P Fig**). We next used quantitative real-time polymerase chain reaction (qRT-PCR) to measure *GRF9* expression in different tissues and observed expression in leaves, flowers and roots; notably, expression in young leaves was higher than in the other tissues tested (**S2A Fig**). Expression of *GRF9* in young leaves indicates a likely role in cell proliferation as shown for other GRFs [22–24]. Similarly, an analysis of previously reported transcriptome data obtained using tiling microarrays for third leaves of Arabidopsis at days 8–14 revealed high expression of *GRF9* at days 8 and 9 when cell proliferation is high, but a decline of expression at day 10 when cell elongation starts (Table S1 in [13]). Thus, *GRF9* transcript level follows the transcriptional activity pattern we observe here for *Pro*_*GRF9*_:*GUS* seedlings.

Phytohormones, in particular auxin and cytokinin, affect cell proliferation [46–50]. We therefore tested the effect of auxin (applied as 2,4-D) and cytokinin (applied as zeatin) on *GRF9* expression by qRT-PCR in 14-day-old wild-type (WT) seedlings treated with different concentrations of these phytohormones, but *GRF9* expression level was not significantly altered in the conditions tested (**S2B Fig**). We also tested the effect of the hormones using *Pro*_*GRF9*_:*GUS* lines, but did not observe a major effect (**S2C Fig**). These results indicate that GRF9 function is likely to be independent of auxin and cytokinin pathways as previously also reported e.g. for *GRF7* [51].

### *grf9* mutants and *GRF9* overexpressors have altered leaf size due to changes in cell number

**Fig 1:**
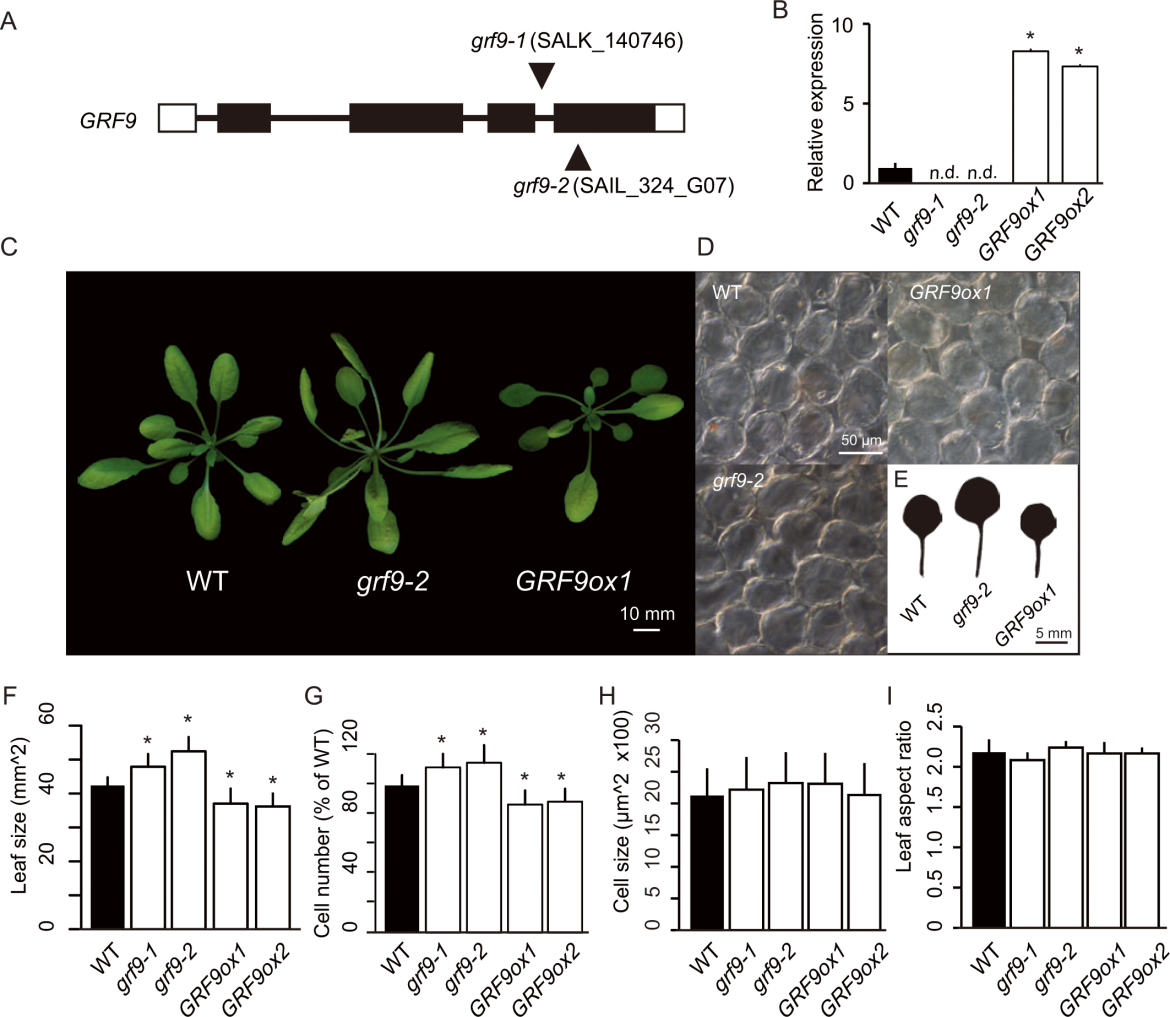
Characterization of the *grf9* and *GRF9ox* lines. (A) Schematic representation of the *GRF9* locus and the locations of the T-DNA insertions (black triangles) in the *grf9-1* and *grf9-2* knockout mutants. White rectangles represent untranslated regions (UTRs), black rectangles show protein-coding regions, and thick connecting lines indicate introns. (B) Expression level of *GRF9* determined by qRT-PCR in *grf9* knockout and *GRF9ox* plants, compared to WT, for which expression is set to 1. No *GRF9* expression was detected in *grf9* knockout mutants (n.d.). The results are shown as means of three replicates ± SD. (C) Rosette phenotype. (D) Palisade cells of first-pair leaves of 21-day-old plants observed from a paradermal view. (E) Scans of representative first-pair leaves of WT, *grf9-2* and *GRF9ox1* seedlings. (F) Leaf sizes of WT, *grf9* mutants and *GRF9ox* lines (n > 8 leaves). (G) Total number of palisade cells in the subepidermal layer of mature first leaves (n > 8 leaves). (H) Sizes of palisade cells observed from a paradermal view (n > 240 cells from more than eight leaves). (I) Leaf aspect ratio (ratio of leaf length to leaf width) of WT, *grf9* mutants and *GRF9ox* plants. Means ± SD. Plants were grown for 3 weeks under a 16 h light / 8 h dark fluorescent illumination cycle at 120 μmol m^-2^ s^-1^. Asterisks in panels B, F and G indicate significant difference (Student's *t*-test; *p* < 0.05) from WT. Bars = 10 mm (panel C), 50 μm (panel D), and 5 mm (panel E).

To identify the biological role of GRF9, we selected two independent homozygous T-DNA insertion lines (*grf9-1*; SALK_140746; *grf9-2*; SAIL_324_G07; **Fig 1A and 1B; S3 Fig**) and *Pro*_*35S*_:*GRF9* over-expression (*GRF9ox1* and *GRF9ox2*) plants for further analyses (**Fig 1B**). Considering the prominent expression of *GRF9* in young leaves, we characterized the leaf phenotypes of the *grf9* mutants and *GRF9ox* lines. The loss-of-function *grf9-1* and *grf9-2* mutants developed enlarged rosettes with larger leaves compared to WT (114% and 123%, respectively; **Fig 1C, 1E and 1F; S4A to S4C Fig**) and a significantly increased cell number (112% and 115%; **Fig 1G**) while cell sizes within leaves remained unchanged (104% and 105%; **Fig 1D and 1H**). In contrast, *GRF9ox1* and *GRF9ox2* plants developed smaller leaves (87% and 85% of WT, respectively) with a decreased cell number (87% and 88%, respectively), while cell sizes were not affected (108% and 102%, respectively; **Fig 1C–1H**). In both *grf9* mutants and the two *GRF9ox* plants, the leaf aspect ratio, defined as the leaf length over the leaf width, was close to that of the WT (**Fig 1I**). Collectively, the negative effect of GRF9 on leaf growth was observed in different photoperiods, namely in long-day (**Fig 1C and 1F**) as well as in short-day and equal-day conditions (**S4A to S4C Fig**).

**Fig 2:**
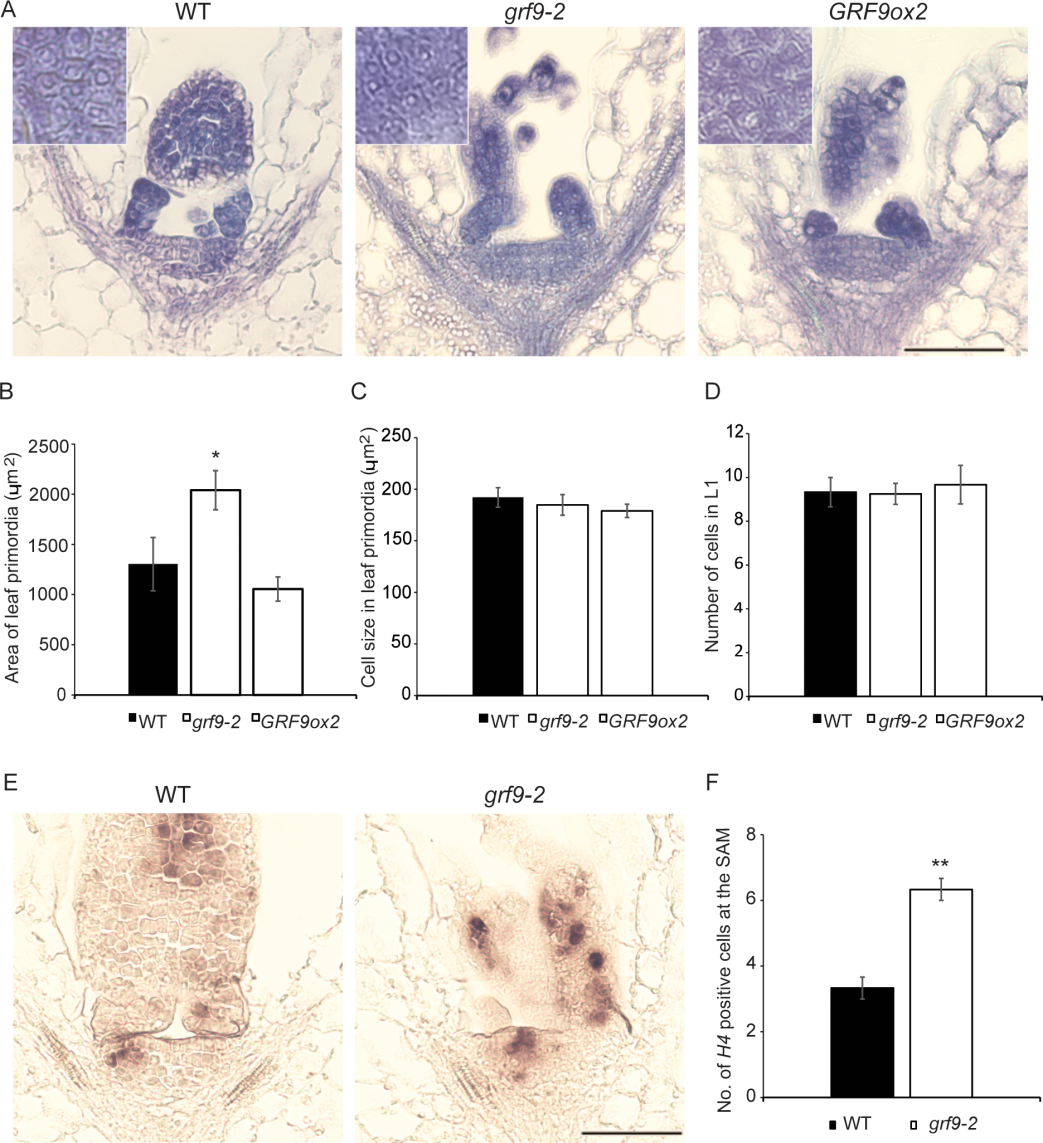
GRF9 affects the size of leaf primordia in the shoot apical meristem (SAM). (A) Emergence of first leaf primordia in 2-day-old WT, *grf9-2* and *GRF9ox2* plants grown in long day (LD) conditions (16 h light/8 h dark) analysed by toluidine blue staining. (B) Area of first leaf primordia of WT, *grf9-2* and *GRF9ox2* plants (n > 4 primordia). (C) Size of cells in leaf primordia of WT, *grf9-2* and *GRF9ox2* plants (n > 7 cells from more than four primordia). (D) Number of cells in the layer 1 (L1) of the SAM (n = 3 plants). (E) RNA *in situ* hybridization using *HISTONE4* (*H4*) as probe on longitudinal sections of the apical meristem with young leaf primordia of 2-day-old WT and *grf9-2* plants. (F) Number of cells expressing *HISTONE4* (*H4*) at the SAM (n = 3 meristems). Error bars represent ± SEM. Scale bars 100 μm (panels A and E).

To determine whether *GRF9* affects leaf size at early stages of development, we embedded shoot apices of 2-day-old seedlings in paraffin and after sectioning analysed the size of incipient leaf primordia emerging from the SAM. As shown in **Fig 2A and 2B**, leaf primordium size was significantly increased in the *grf9-2* mutants, but trended to be reduced in *GRF9ox2* seedlings, compared to wild type. However, cell sizes in leaf primordia did not differ significantly between the genotypes (**Fig 2C**), indicating that more cells than in the WT contribute to the bigger primordia in *grf9* seedlings, while the opposite appears to happen in *GRF9* overexpressors. Of note, the size of the SAM–represented by the number of cells in the L1 layer—did not differ between the different genotpyes (**Fig 2D**), indicating that cell proliferation was more active in the incipient leaf primordia of *grf9-2* than WT plants, but lower in those of *GRF9ox2* plants. This conclusion is supported by a considerably higher expression of the G1-S phase cell cycle marker gene *HISTONE4* (*H4*) in the leaf primordia and the SAM of *grf9-2* compared to WT, as revealed by RNA *in situ* hybridization (**Fig 2E and 2F**). In addition, we analyzed the transcripts of another cell cycle activity gene, *CYCLIN B1;1* (*CYCB1;1*), by RNA *in situ* hybridization. Similar to *H4*, we found higher *CYCB1;1* transcript levels in the SAM of *grf-2* mutant plants than the wild-type (**S5 Fig**).

We also characterized the petal phenotype of *grf9* mutants and *GRF9ox* plants. Notably, petal size was significantly increased in both, the *grf9-1* and *grf9-2* mutants (125% and 117%, respectively; **S6A and S6B Fig**) while the size of the petal cells remained unaltered (95% and 103%, respectively; **S6C Fig**). On the contrary, *GRF9ox1* plants exhibited smaller petals with normal cell sizes (**S6A to S6C Fig**). Taken together, these results suggest that *GRF9* contributes to determining final organ size by limiting cell proliferation during plant development.

### Cell proliferation kinetics in developing leaves of *grf9* and *GRF9ox* plants

**Fig 3:**
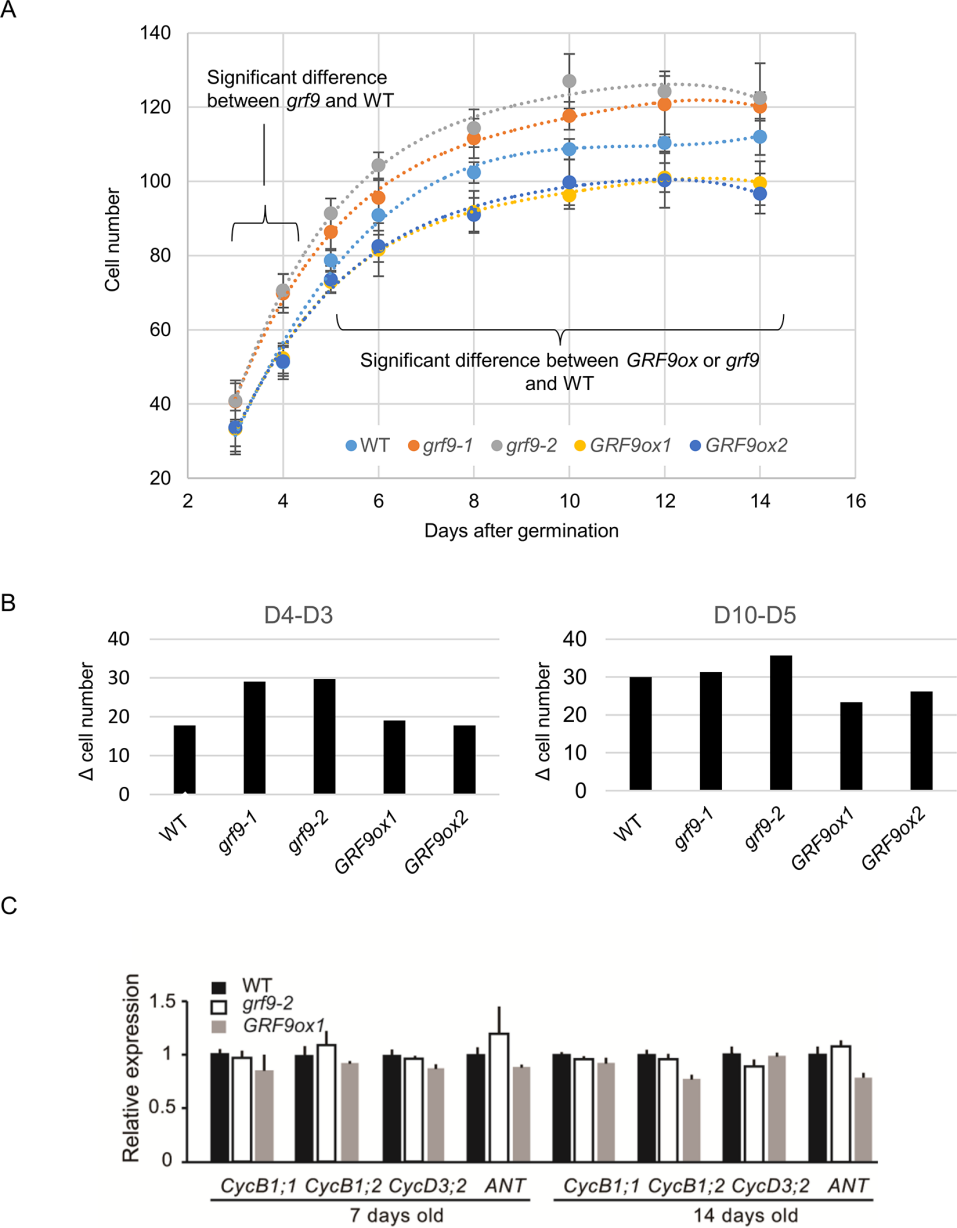
Time-course analysis of leaf growth. (A) Number of cells along the basal-apical axis of the first set of developing leaves in WT, *grf9* knockout and *GRF9ox* lines. The curves show fitting to the experimental data with a polynomial function. R^2^ values are > 0.99 in all cases. At days 3 and 4 after germination, cell numbers are significantly higher in the two *grf9* mutants than in WT (Student's *t*-test; day 3, *p* ≤ 0.01; day 4, *p* ≤ 0.001). At days 5 to 14, cell numbers are significantly higher in the two *grf9* mutants than in WT, and significantly lower in the two *GRF9ox* lines (Student's *t*-test; *P* values between *p* ≤ 0.05 and *p* ≤ 0.001). Data are means of 6–14 leaves for the different genotypes and time points ± SD. The full data are given in **S4 Table**). (B) Increment of the number of cells in the adaxial subepidermal layer along the basal-apical leaf axis. Left: increment (Δ cell number) between day 3 and day 4 (D4-D3); right: increment between day 5 and day 10 (D10-D5) in WT and *GRF9* transgenic plants. See **S4 Table** for the full data. (C) Relative expression levels of cell cycle-related genes in the leaf primordia from 7-day-old and 14-day-old WT, *grf9-2* and *GRF9ox1* plants. Gene expression in the WT is set to 1. Data are means ± SD (n > 8 seedlings).

As GRF9 affects cell proliferation already in the incipient leaf primordium, we wanted to know whether it also affects cell numbers subsequently, when leaves develop further. To address this, we performed a kinematic analysis of cell proliferation in young developing leaves (**Fig 3**). Cell number was determined by counting the number of adaxial subepidermal cells along the length of the first pair of leaves [11]. As shown in **Fig 3A**, cell number increased in seedlings of all genotypes until day 10 to 12 and then did not change further thereafter. Cell numbers were significantly higher throughout the entire observation period in the two *grf9* knockout mutants compared to WT, in accordance with a significantly bigger incipient leaf primordium in *grf9* (see **Fig 2B**). Cell numbers were not significantly different between *GRF9ox* and WT seedlings at days 3 and 4 after germination, but were significantly lower in the overexpressors than the WT from day 5 onwards. We calculated the rate with which cell number increased during leaf development. As shown in **Fig 3B**, between days 3 and 4, cell numbers increased more in *grf9* knockout mutants than in WT, while there was no detectable difference between WT and *GRF9ox* lines. At later stages of leaf development (from day 5 to day 10), cell numbers increased less prominently in *GRF9ox* seedlings than WT.

We next assessed the expression of cell cycle marker genes: *CYCB1;1* (*CYCLIN-B1;1*, B-class cyclin gene) and *CYCB1;2*, *CYCD3;2* (*CYCLIN-D3;2*, D-class cyclin gene), and *ANT* (*AINTEGUMENTA*) [22,52–54] in developing leaves of 7- and 14-day-old seedlings, by qRT-PCR. We observed that expression of *CYCD3;2*, which encodes a key regulator of integrating cell division in lateral organ development [52,53], was similar in *grf9*, *GRF9ox* and WT in both, 7-day-old and 14-day-old plants (**Fig 3C**). We observed the same for *CYCB1;1*, *CYCB1;2*, and *ANT*, which function in cell proliferation and organ growth throughout plant development, supporting the model that cell proliferation rate in the later stages of developing leaves was not overtly affected in *grf9* and *GRF9ox* plants.

### *grf9* mutants have an enhanced cell proliferation within the developing leaf

**Fig 4:**
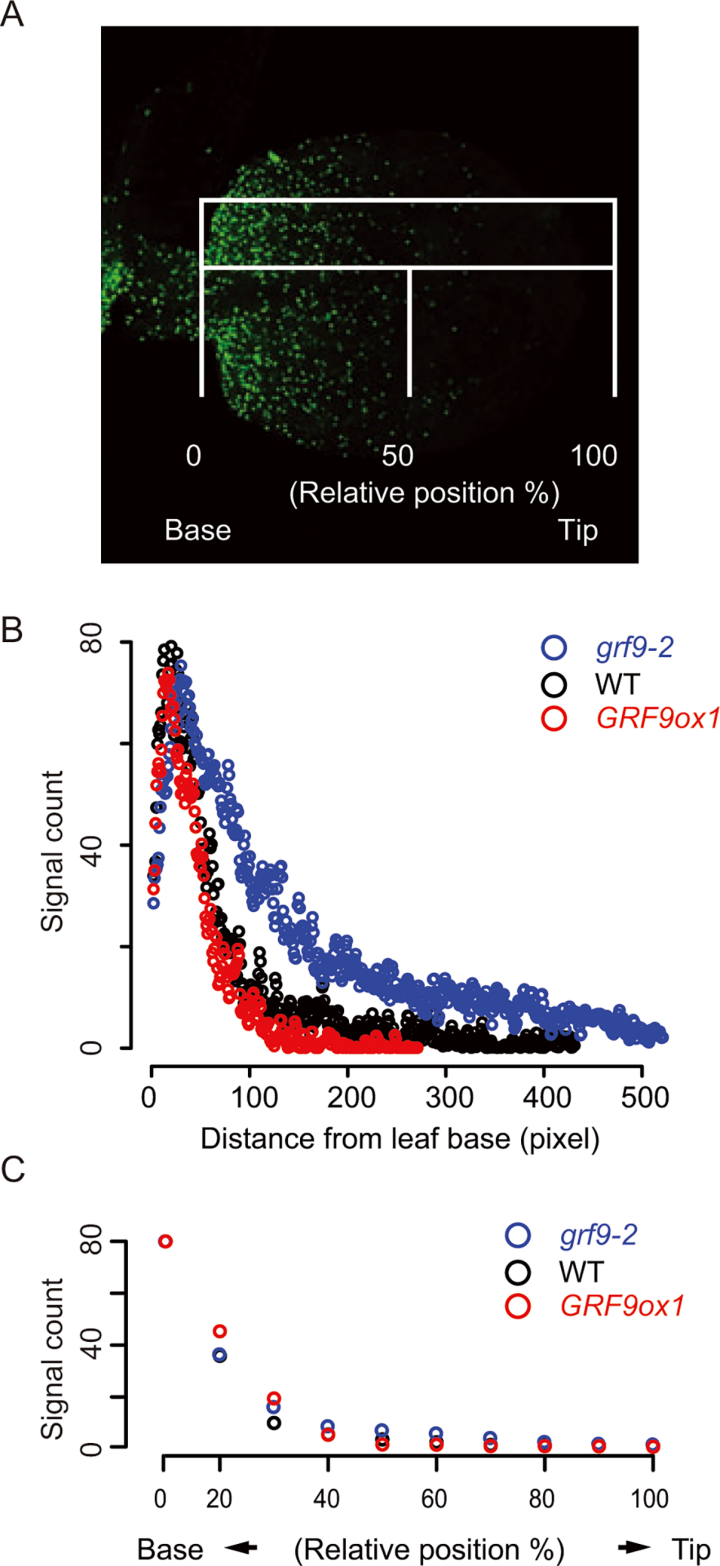
Determination of actively proliferating cells in WT, *grf9* and *GRF9ox* plants using 5-ethynyl-2'-deoxyuridine (EdU) incorporation assay. The first true leaves of 5-day-old seedlings were analysed. (A) Example of an EdU-stained Arabidopsis leaf. Green signals indicate cells undergoing mitosis. (B) EdU signal distribution in leaves, and (C) relative occupancy of proliferating cells within the developing leaves in *grf9-2*, *GRF9ox1* and WT plants. Data represent average signals from at least eight seedlings.

It has been reported that some GRFs and their interaction partners, such as GIF1 and the transcription factor TCP4, as well as *miR396*, are involved in controlling the progression of the AF, the boundary separating the cell proliferation from the cell differentiation area in the developing leaves [15,16,22,33,42,43,55]. Considering that GRF9 affects the size of the incipient leaf primordium and that cell number was affected in young leaves we considered the possibility that the area occupied by proliferating cells in the developing organ was changed in *grf9* mutants. To test this, we visualized the proliferating cells in young leaves. One of the most accurate ways of distinguishing actively dividing from non-dividing cells is to directly detect DNA synthesis [56,57]. We therefore used the 5-ethynyl-2'-deoxyuridine (EdU) incorporation assay, which allows visualising S phase cells in animal and plant cells [44,56,58–61]. As expected, EdU signal is higher in the basal parts of young leaves where massive cell proliferation occurs, and the signal fades towards the distal part of the leaf (**Fig 4A**).

More than eight observations revealed that the number of cells having detectable EdU signal was considerably increased in *grf9* plants compared to WT. In contrast, *GRF9ox* plants showed a slight although insignificant decrease in the number of cells compared to WT (**Fig 4B**). We also determined the relative occupancy of the cell proliferation area relative to the total area of the leaf primordia in *grf9*, *GRF9ox* and WT plants, but did not detect significant differences between them (**Fig 4C**).

### GRF9 binding site identification

**Fig 5:**
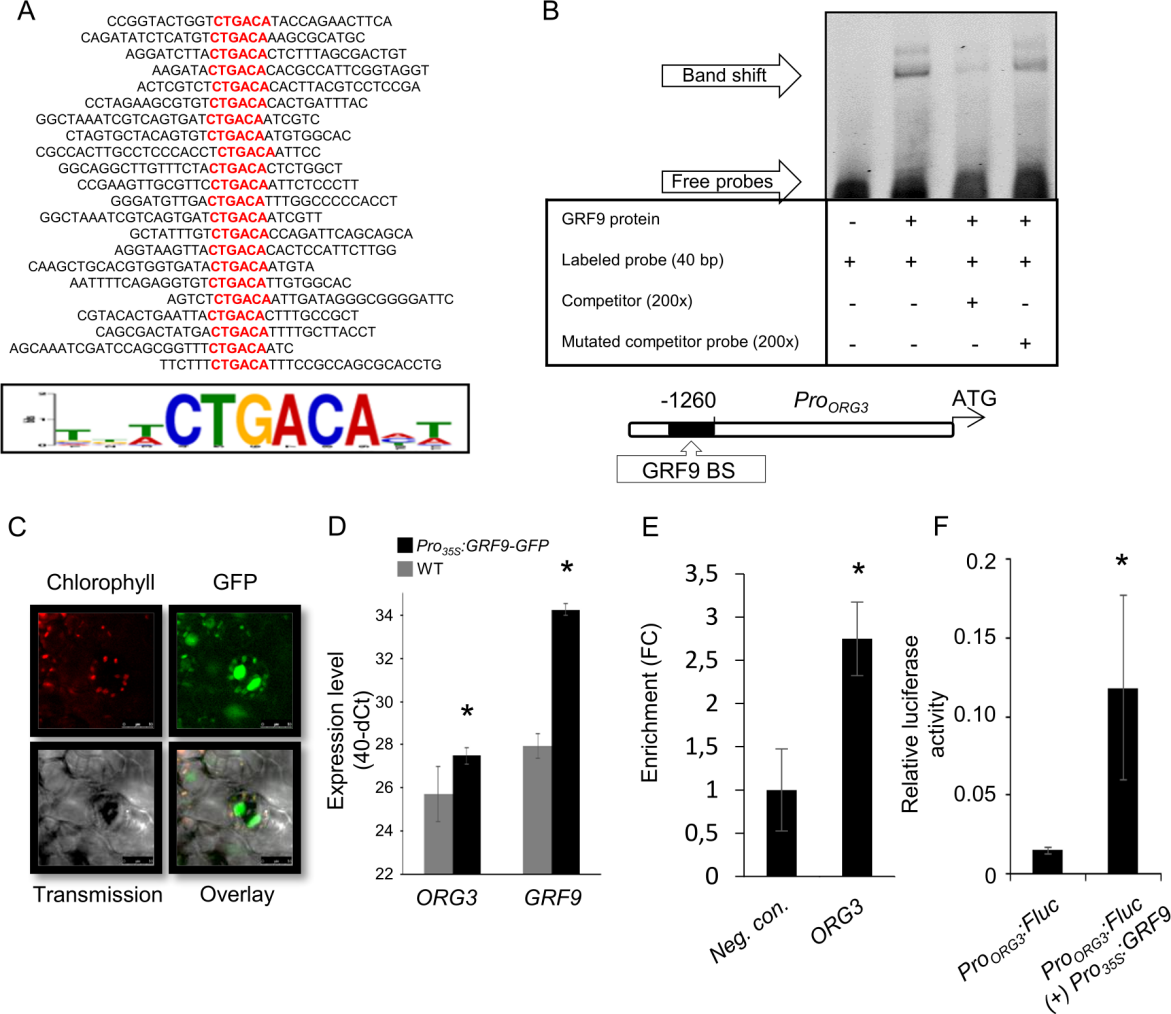
GRF9 directly regulates *ORG3*. (A) GRF9 DNA-binding sequences determined using *in vitro* binding site selection. All selected oligonucleotides contain a functional GRF9-binding site as verified in DNA-binding assays. The nucleotides in the core binding sequence are shown in red. The logo of the GRF9 binding sequence profile was generated using the MEME program (http://meme.sdsc.edu/meme/cgi-bin/meme.cgi). (B) GRF9 binds *in vitro* to the *ORG3* fragment harboring the GRF9 binding site. A schematic view of the *ORG3* promoter (around 1.5 kb upstream of the translation start site) containing the GRF9 binding sequence (BS) is shown at the bottom (BS; black box). Electrophoretic mobility shift assay (EMSA) using GRF9-CELD protein and a 40-bp sequence of the *ORG3* promoter harboring the GRF9 BS. GRF9-CELD protein incubated with those oligonucleotides causes retardation ('Band shift'). Retardation disappeared in the presence of competitor (unlabeled probe at high concentration) whilst adding a molar excess of mutated probe did not block the interaction between GRF9 protein and the labelled probe, indicating specific binding of GRF9 to the CTGACA binding site. (C) Laser scanning confocal microscopy images showing nuclear localization of GRF9-GFP fusion protein in 3-week-old transgenic Arabidopsis plants expressing GFP-tagged GRF9 protein. (D) Expression level of *GRF9* and *ORG3* in *Pro*_*35S*_:*GRF9-GFP* and WT (Col-0) plants. Expression was determined by qRT-PCR and values represent the means of replicates from three biological replicates ± SD. (E) GRF9 binds *in vivo* to the *ORG3* promoter. ChIP-qPCR results of 5-day-old *Pro*_*35S*_:*GRF9-GFP* Arabidopsis seedlings. Data represent average enrichment (fold change, FC) in three independent biological replicates ± SD. (F) GRF9 transactivates the *ORG3* promoter *in vivo*. Relative luciferase activity detected in Arabidopsis mesophyll cell protoplasts. Data are means ± SD of three independent transformations, each representing five technical replicates. The asterisks indicate significant difference (Student's *t*-test; *p* < 0.05).

Several GRF transcription factors affect leaf growth by regulating cell proliferation [21–24,29,62]. However, little is known about the downstream targets of these TFs and their gene regulatory networks. Therefore, to identify potential targets of GRF9, we first determined its binding site by *in vitro* binding site selection assay using the CELD fusion protein method [63]. We identified the 6-base motif 5'-CTGACA-3' as the core GRF9 binding site (**Fig 5A**). Mutational analysis revealed that altering individual nucleotides within the identified core binding site dramatically diminished GRF9 binding activity (**S7 Fig**).

### Genes affected by GRF9

We next identified genes affected by GRF9, employing transgenic Arabidopsis plants expressing *GRF9* from an estradiol (EST)-inducible promoter (hereafter, *GRF9-IOE* plants). We induced *GRF9* expression by EST treatment for 3 and 4 h (using 2-week-old *GRF9-IOE* seedlings) and 6 h (using detached mature leaves from 4-week-old soil grown *GRF9-IOE* plants) and performed transcriptome profiling using Affymetrix ATH1 microarrays. We compared all three transcriptome data sets with those of the controls (mock treated with 0.15% ethanol) and identified 89 genes upregulated and five genes downregulated after EST treatment in at least two of the three time points (by at least 2-fold) (**S1 Table**). Twenty-three upregulated genes (including *GRF9* itself) harbour at least one GRF9 binding site within their 1.5-kb promoters (**S1 Table**), identifying them as potential downstream targets of GRF9. Enhanced expression of most of the 23 genes was confirmed by qRT-PCR in independent biological samples of EST-induced *GRF9-IOE* seedlings (different induction times) as well as in the overexpression line *GRF9ox1*, while their expression was reduced in the *grf9-1* knockout mutant (**S8 Fig**). Of note, expression of other *AtGRFs* and *miR396* was not significantly altered after EST induction of *GRF9* in the *GRF9-IOE* line, or in different *GRF9* transgenic plants, respectively (**S1 Table**).

Based on their expression levels, harbouring of GRF9 binding site and literature review we selected *OBP3-RESPONSIVE GENE 3* (*ORG3*), also known as *bHLH039*, as a potential target gene of GRF9 for further analysis.

### GRF9 binds the *ORG3* promoter *in vitro*

Regulation of gene expression involves the direct interaction of transcription factors with *cis*-regulatory elements located in the promoters of target genes. To investigate the physical interaction of GRF9 with the *ORG3* promoter, we performed electrophoretic mobility shift assays (EMSAs). Sequence analysis of the *ORG3* promoter (1.5 kb upstream of the translation start site) revealed the presence of one full-length GRF9 binding site (5'-CTGACA-3'; called BS in the following) ~1.3 upstream of its translation start site (**Fig 5B**). We tested interaction of recombinant GRF9 protein with a 40-bp long, 5'-DY682-labeled double-stranded oligonucleotide harbouring BS. As shown in **Fig 5B**, the DNA-protein complex migrated more slowly than free DNA indicating direct interaction of GRF9 with the labelled DNA. When unlabelled competitor DNA (oligonucleotide containing binding site) was added in molar excess, a strong reduction in signal intensity was observed; moreover, molar excess of the mutated version of the BS did not diminish binding to the correct BS (**Fig 5B**), confirming specificity of the interaction.

### *In vivo* binding of GRF9 to the *ORG3* promoter

To test whether GRF9 interacts *in vivo* with the *ORG3* promoter, we performed chromatin immunoprecipitation (ChIP) and tested the enrichment of *ORG3* promoter fragments by quantitative PCR (ChIP-qPCR), using transgenic Arabidopsis lines expressing GRF9-GFP fusion protein from the CaMV *35S* promoter. As shown in **Fig 5C**, GRF9-GFP fusion protein accumulated in the nuclei of transgenic plants, consistent with its role as a transcription factor. In addition, *ORG3* expression was elevated compared to WT in *Pro*_*35S*_:*GRF9-GFP* lines (**Fig 5D**), indicating that the GRF9-GFP fusion protein activated *ORG3* similar to GRF9. Using these plants, we observed a significant enrichment of the *ORG3* promoter region harbouring the GRF9 binding site (**Fig 5E**), supporting the conclusion that it is a direct downstream target of GRF9.

### Transactivation of the *ORG3* promoter in mesophyll cell protoplasts

To test whether *ORG3* is transactivated by GRF9, we performed luciferase-based transactivation assays using Arabidopsis mesophyll cell protoplasts (**Fig 5F**). A reporter construct containing the firefly luciferase (FLuc) coding region under the control of the ~1.5-kb *ORG3* promoter (*Pro*_*ORG3*_:*FLuc*) (**Fig 5F**) was transformed into protoplasts in the presence or absence of *Pro*_*35S*_:*GRF9* effector plasmid. A significantly higher luciferase activity was observed when *Pro*_*ORG3*_:*FLuc* was co-transformed with *Pro*_*35S*_:*GRF9* than in controls that were only transformed with the *Pro*_*ORG3*_:*FLuc* construct, indicating that GRF9 transactivates *ORG3* expression in mesophyll cell protoplasts (**Fig 5F**). In the absence of *Pro*_*35S*_:*GRF9*, only low basal luciferase activity was observed (**Fig 5F**).

### *ORG3* restricts organ size

**Fig 6:**
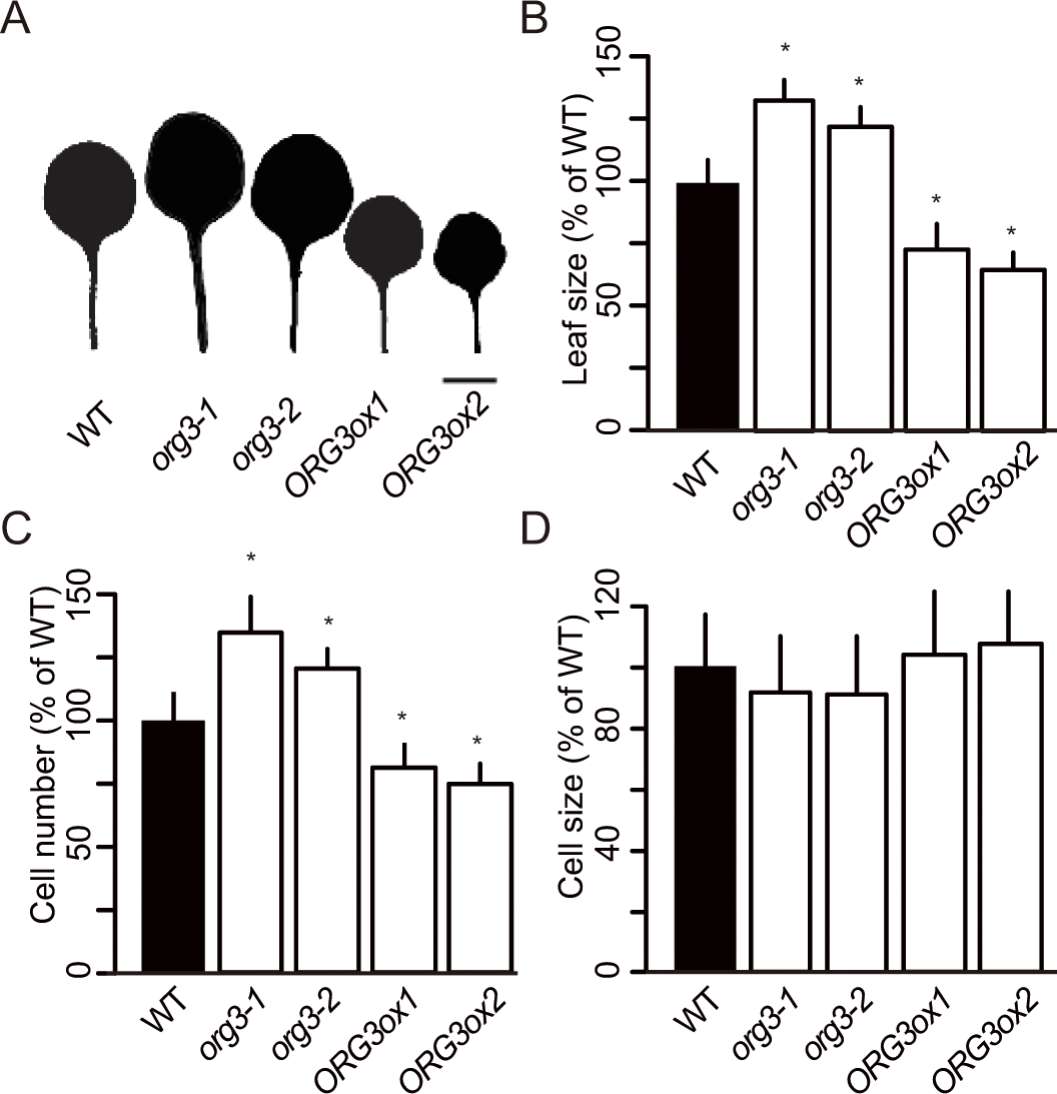
Characterization of *org3* mutants and *ORG3ox* plants. (A) Scans of representative first-pair leaves, (B) leaf size, (C) number of palisade cells, and (D) size of palisade cells (n > 240 cells) in WT, *org3-1*, *org3-2*, *ORG3ox1* and *ORG3ox2* plants. Results are expressed as percentage of WT ± SD. First-pair leaves from 21-day-old plants were analyzed (n > 8 in all cases). Asterisks indicate significant difference from WT (Student's *t*-test; *p* < 0.05). In (A), bar = 10 mm.

Our above results demonstrate that GRF9 positively regulates *ORG3* expression by binding to its promoter, suggesting that GRF9 affects the cell proliferation process in developing leaves through *ORG3*. To address this point further, we characterized the impact of *ORG3* on leaf development. First, we determined expression of *ORG3* in two *org3* knockout mutants (*org3-1* and *org3-2*), both harbouring T-DNA insertions in the first exon (**S9A and S9B Fig**). Endpoint PCR as well as qRT-PCR revealed that accumulation of *ORG3* transcripts in each line was drastically reduced in young seedlings of the two *org3* mutant plants compared to WT (**S9C and S9D Fig**). Interestingly, both, *org3-1* and *org3-2* showed bigger leaves than WT (131% and 122%, respectively) with a significant increase in cell number (143% and 124%, respectively; **Fig 6A–6C**), like the two *grf9* mutants (**Fig 1E–1G**). In contrast, the size of leaf cells was not affected in *org3-1* and *org3-2* mutants (**Fig 6D**). Our data therefore suggest that in leaves the loss of *ORG3* causes a specific defect in cell proliferation. The same phenotype was found in petals in which loss of *ORG3* caused increased petal size (**S10A and S10B Fig**). Cell size measurements showed that the size of the petal cells was not altered in *org3-1* compared to WT (**S10C Fig**), indicating that the larger petals in *org3* mutant are due to an increase in cell number. To estimate the impact of *ORG3* on plant development, we produced overexpression lines of *ORG3* (hereafter, *ORG3ox1* and *ORG3ox2*) (**S9D Fig**). As a consequence, *ORG3ox1* and *ORG3ox2* plants showed significantly smaller leaves (75% and 68%, respectively) than the WT with a specific defect in cell proliferation (**Fig 6**). These results suggest that *ORG3* restricts organ size by limiting cell number.

### *ORG3* acts downstream of GRF9

**Fig 7:**
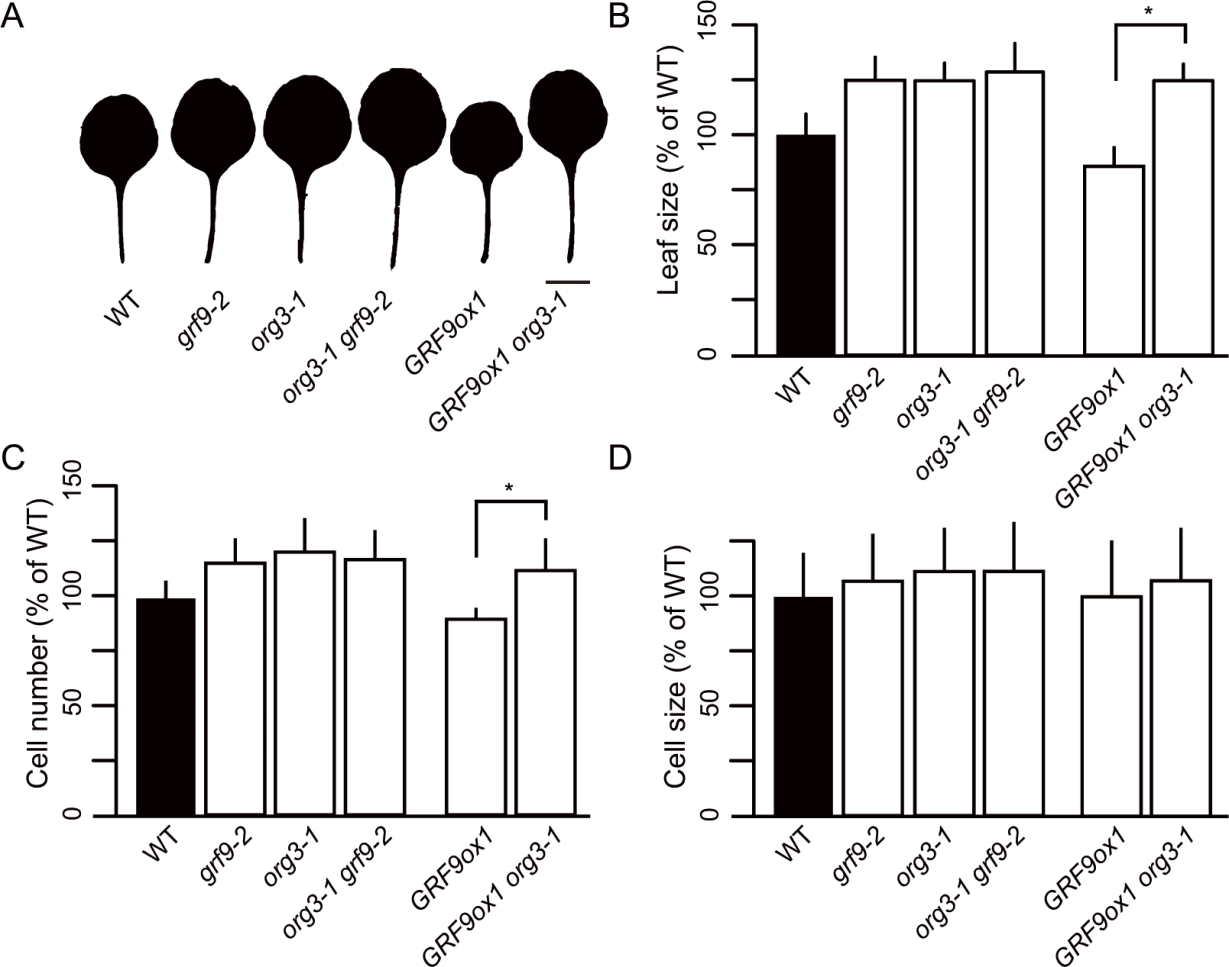
Characterization of the *grf9 org3* and *GRF9ox org3* double mutants. (A) Scans of representative first-pair leaves, (B) leaf area, (C) number of palisade cells, and (D) cell area of palisade cells (n > 240 cells) in WT, *grf9-2*, *org3-1*, *org3-1 grf9-2* (line 7), *GRF9ox1*, and *GRF9ox1 org3-1* (line 34) mutants. Data are expressed as a percentage of WT ± SD. First leaves from 21-day-old plants were analyzed (n > 8 in all cases). Asterisks in panels (B) and (C) indicate significant difference (Student's *t*-test; *p* < 0.05). In (A), bar = 10 mm.

The molecular data reported in the previous sections, as well as the similar growth phenotypes observed in *GRF9* and *ORG3* overexpressors (reduced leaf sizes), and in *grf9* and *org3* knockout mutants (increased leaf sizes) suggested that GRF9 and *ORG3* form a regulatory cascade in leaf development. To further substantiate this model, we created *grf9-2 org3-1* and *GRF9ox1 org3-1* double mutants (**Fig 7, S9E and S9F Fig**). We reasoned that simultaneously knocking out both genes would further increase leaf size (over the sizes of the single-gene mutants) if both transcription factors acted in independent biological pathways. However, as shown in **Fig 7A–7D**, leaf sizes, cell numbers and cell sizes of the *grf9-2 org3-1* double mutant were not significantly different from those of the parent single-gene mutants, suggesting that *ORG3* acts in the same pathway as *GRF9* to regulate leaf development. Notably, while overexpression of *GRF9* in the wild-type Col-0 background (*GRF9ox* plants) leads to reduced leaf size and cell number (**Fig 1F and 1G; Fig 7A–7C**), this effect is entirely lost when *GRF9* is overexpressed in the *org3-1* knockout mutant (**Fig 7A–7C**). Taken together, our data confirm a genetic interaction between *GRF9* and *ORG3*, whereby *ORG3* acts downstream of GRF9 to determine leaf size.

## Discussion

Final leaf size has a major effect on photosynthetic performance and the formation of biomass [1]. Leaf growth and development are controlled by a complex network of genes in which TFs are important controllers. In this study, we addressed the function of the transcription factor GRF9 for leaf growth in Arabidopsis. GRF9 belongs to the plant-specific GROWTH-REGULATING FACTOR (GRF) family, which in Arabidopsis includes nine members playing important biological roles [21–24,38,40,41,51,64,65].

Using *Pro*_*GRF9*_:*GUS* reporter lines, we observed *GRF9* reporter activity e.g. in the proliferation zone of developing leaves, similar to other members of the *GRF* family [22–24,34,66]. The transcriptional activity conferred by the *GRF9* promoter broadly mirrored *GRF9* expression determined in transcriptome studies using tiling arrays: *GRF9* expression was high in leaves with proliferating cells while it gradually decreased when elemental expansion, also known as surface extension [10], became prominent [13]. *GRF9* is also expressed in flowers (here mostly in the carpels), the abscission zone of siliques, and in roots. In these tissues as well as in developing leaves, *GRF9* expression might be finely tuned by *miR396* (see below).

### *GRF9* restricts cell proliferation in the incipient leaf primordium

**Fig 8:**
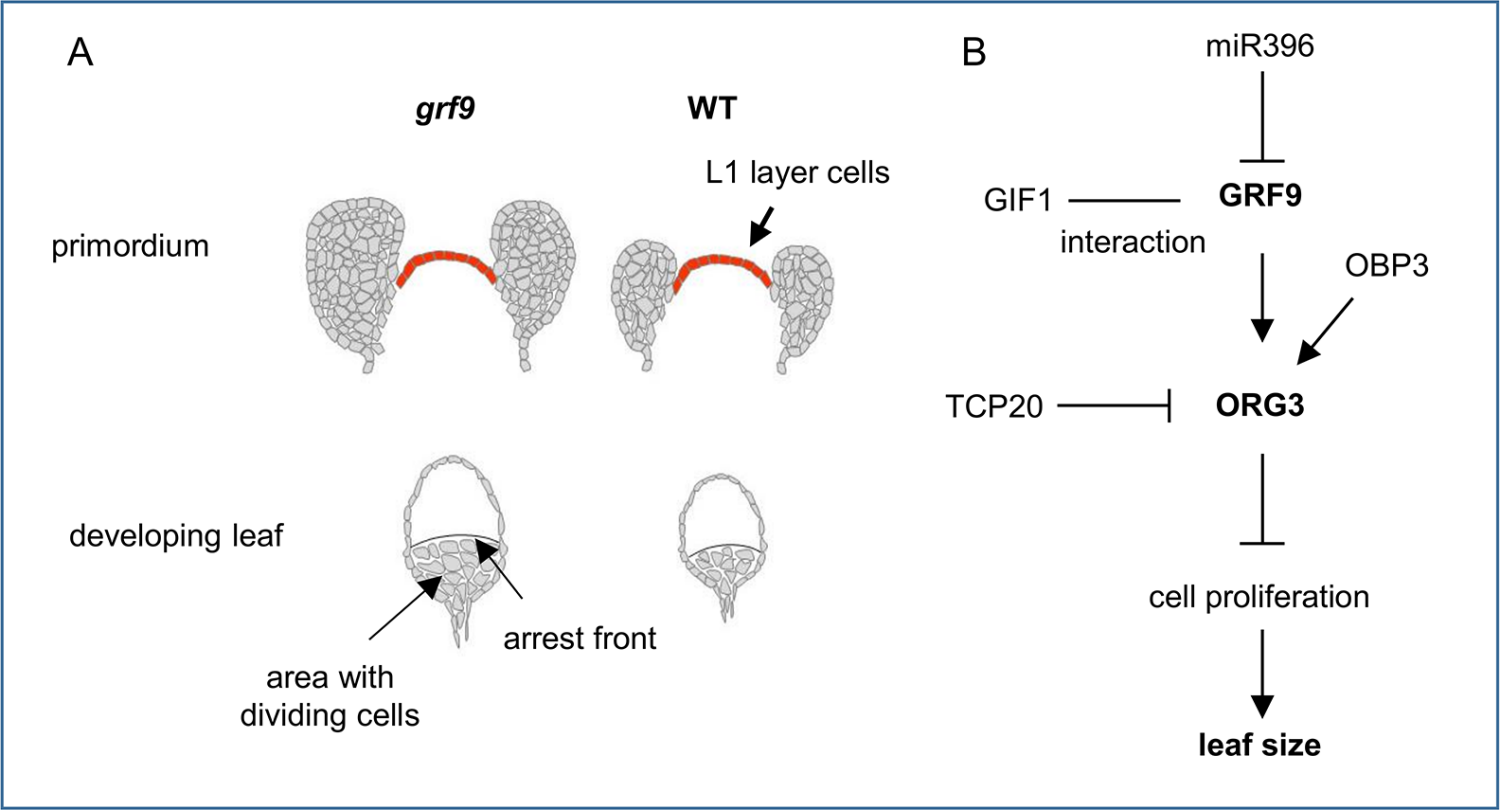
Model of GRF9 action. (A) GRF9 restricts the size of the incipient leaf primordium at the shoot apex, whitout affecting cell size or the size of the SAM. While leaf primordium size is increased in *grf9* mutants, compared to wild type (WT), it trends to be smaller in *GRF9* overexpressor (*GRF9ox*) plants. Similarly, cell numbers in young developing leaves are bigger in *grf9*, but smaller in *GRF9ox* plants, in accordance with a higher number of cells in the developing *grf9* leaves, potentially contributing to the position of the arrest front. (B) Gene regulatory network by which GRF9 and ORG3 influence leaf size. *MiR396* targets *GRF9* transcript and negatively regulates its abundance. GRF9 interacts with GIF1 which, similar to other GRFs, influences leaf size determination. *ORG3* expression is positively regulated by GRF9 and OBP3, but repressed by the TCP20 transcription factor [7]. Finally, ORG3 negatively regulates cell proliferation thereby directly influencing leaf size. For more details, see text.

We employed reverse genetics approaches to reveal the functional importance of *GRF9* in leaf development. Loss-of-function *grf9* mutants had bigger rosette leaves and petals than WT while plants overexpressing *GRF9* produced smaller leaves and petals by affecting cell numbers but not cell sizes. Bigger leaves were previously reported for one of the *grf9* mutants studied here (*grf9-1*) and the phenotype of it observed in this study is in accordance with previous results [22,67], although Horiguchi *et al*. (2005) [22] did not find the increase in leaf size in *grf9* to be significant. Although several members of the *GRF* family in Arabidopsis positively determine final leaf size by affecting cell proliferation or elemental expansion [21–24], we demonstrate here that *GRF9* negatively regulates leaf size dimensions by inhibiting cell proliferation in the incipient leaf primordium and the developing leaf which might affect the position of the cell cycle arrest front (see **Fig 8**). The increased size of the incipient leaf primordium in the *grf9-2* mutant occurred without a change in the size of SAM or cell sizes, suggesting a higher cell proliferation rate in *grf9-2* during the initial phase of leaf establishment which is supported by a considerably higher expression of *HISTONE4* and *CYCB1;1* in *grf9-2* than WT primordia (**Fig 2E and 2F and S5 Fig**).

One of the most accurate ways of identifying actively proliferating cells is to directly label newly synthesized DNA in the dividing cells e.g. using 5-ethynyl-2'-deoxyuridine (EdU) which has a structure similar to that of thymidine thereby facilitating its incorporation into newly synthesized DNA molecules [56,58,61]. EdU staining has been successfully employed in Arabidopsis for the determination of actively proliferating cells in the leaf primordium as well as in the quiescent centre (QC) of roots [44,68]. By employing the EdU incorporation assay we here show that *grf9* knockout mutants have significantly more proliferating cells than the wild type, while the number of proliferating cells is slightly reduced in *GRF9ox* lines (**Fig 4**). Thus, *GRF9* determines final leaf size by affecting the number of cells within the incipient leaf primordium and thereafter the number of cells in young leaves. *GRF9* might exert its function by restricting the cell proliferation process in developing leaves or by reducing the number of leaf founder cells which has not been investigated here.

It has been suggested that *miR396*, which inhibits various *GRFs*, contributes to the movement of the AF in the developing leaf [16]. *Vice versa*, GRFs can regulate *miR396* expression [69]; one may, therefore, speculate that overexpression of *GRF9* can synergistically regulate *miR396* expression and antagonistically regulate other *GRFs*, resulting in fewer cells in the developing leaf. Furthermore, as *GRF9* harbours a GRF9 binding site in its promoter, there is the further possibility that it autoregulates its own expression; whether this is indeed happening was however not tested here.

Previously, Wu *et al*. (2014) reported that *ZmGRF10* (*Zea mays GRF10*) negatively regulates cell numbers and leaf size in maize [34]; interestingly, *ZmGRF10* belongs to the same phylogenetic class as *AtGRF9* but is different from other GRFs which act as positive regulators of leaf size [65]. A model was proposed in which the homeostasis of GRF/GIF (GRF-INTERACTING FACTOR) complexes regulates cell proliferation in maize leaves where this homeostasis is adjusted by ZmGRF10 as a negative, and ZmGRF1 as a positive regulator [34,45]. Horiguchi *et al*. (2005) also showed in Arabidopsis that the GRF5/GIF1 complex is required for establishing a proper leaf size by promoting cell proliferation activity in the leaf primordium [22]. These authors showed, by yeast two-hybrid analysis, that GRF9 interacts with GIF1 and it may therefore be speculated that the same type of regulation exists for GRF9 although it functions as a negative regulator of cell proliferation.

The previous reports as well as our data suggest that GRF9 functions different from the other members of the GRF family in Arabidopsis, which might be due to its unique protein structure harboring two WRC domains while most other GRFs have only one such domain [21,65,70,71]. Currently, the molecular significance of the presence of two WRC domains in some plant GRF proteins remains unknown. Moreover, AtGRFs harbor the conserved QLQ domain which, however, within the Arabidopsis proteins is unique in GRF9 where a leucine is replaced by phenylalanine [21], althougt this does not affect its interaction with GIF1 [22,41]. Interestingly, it has been shown that other members of the GIF family in Arabidopsis, namely GIF2 and GIF3, interact with all GRFs except GRF9 [41]. Whether this is due to the presence of two WRC domains in GRF9 needs to be investigated further. One possible mode of action is that GRF9 contributes to locate the cell cycle arrest front (AF) by controlling the transition from cell proliferation to cell elongation. This model is consistent with the fact that expression of *GRF9* is restricted to the proximal part of the young growing leaf while it fades towards the AF and further supported by the observation that the AF is shifted to a more distal part of the leaf blade when *GRF9* is knocked out.

Using *in vitro* binding site selection, we identified the six-base motif 5'-CTGACA-3' as the core binding site of GRF9 (**Fig 5A**). This binding site resembles that of GRF7 (TGTCAGG) [51]. Another study has shown that a short sequence enriched in CTG or CAG residues contains the binding site for *Oryza sativa* GRF3 (OsGRF3) and OsGRF10 [72]. The authors also showed that GRF4, 5 and 6 from Arabidopsis bind to this short sequence and concluded that those are conserved motifs in mono- and dicots. Our results support this model.

### A GRF9 –*ORG3* regulatory cascade

We identified several potential target genes of GRF9, one of which is *OBP3-RESPONSIVE GENE3* (*ORG3*; **S1 Table**). qRT-PCR analysis revealed downregulation of *ORG3* expression in *grf9* mutants, and enhanced expression in *GRF9* overexpressors (**S8 Fig**). Using a luciferase-based transactivation assay in Arabidopsis mesophyll cell protoplasts, we showed that GRF9 activates *ORG3* (**Fig 5F**). Moreover, *in vitro* (EMSA) as well as *in vivo* (ChIP) analyses revealed direct binding of GRF9 to the *ORG3* promoter (**Fig 5B and 5E**).

Here, in our study, loss-of-function of *ORG3* resulted in bigger rosette leaves with an increased number of cells compared to WT, while cell sizes remained unchanged, similar to *grf9*. Similar to our study, Van Dingenen *et al*. (2017) [73] reported bigger leaves of the *org3-1* (*bhlh39*) mutant, compared to WT, due to an increase in leaf pavement cell number.

To substantiate our finding that knocking out *ORG3* triggers the formation of bigger leaves, we tested a second knockout mutant (*org3-2*) and made the same observation while overexpression of *ORG3* resulted in smaller leaves (**Fig 6**). Our data thus strongly suggest that ORG3, like its upstream regulator GRF9, functions as a negative regulator of leaf growth. These data, together with the genetic interaction studies, demonstrate that GRF9 and *ORG3* establish a previously unknown regulatory cascade to control leaf size (**Fig 8**).

Moreover, norflurazon (NF), a chemical inhibitor of retrograde signaling and chloroplast differentiation, causes both, a delay in the transition from cell proliferation to elemental expansion and differentiation processes; plants treated with NF had an increased cell proliferation area and the position of the arrest front was closer to the leaf tip [13], similar to what we observed here for the *grf9* mutant. Of note, expression of *ORG3* is strongly reduced in NF-treated plants. In untreated plants, *ORG3* shows maximal expression at days 9 and 10, i.e. when the transition of cell proliferation to elemental expansion occurs, while expression decreases thereafter, in accordance with the model that ORG3 contributes to establishing the cell cycle arrest front during leaf development [7].

### Stress- and ABA-related genes are among the GRF9 early responsive genes

Gene expression profiling revealed a high number of stress- and ABA-related genes among the genes differentially expressed upon induction of *GRF9* expression (**S1 Table**). It has been reported that other members of this family, including *GRF1*, *GRF3* and *GRF7*, play important roles in the coordination of plant growth with stress responses [38,39,51]. We checked the list of differentially expressed genes in the *GRF9-IOE* datasets and publically available transcriptome data from plants overexpressing *miR396*-resistant versions of *GRF1* (*rGRF1ox)* and *GRF3* (*rGRF3ox*), both producing bigger leaves than WT like the *grf9* mutants, as well as of the *grf1*/*2*/*3* triple mutant, producing smaller leaves [38], like *GRF9* overexpressors. While only two genes repressed in *GRF9-IOE* where induced in *rGRF1ox* or *rGRF3ox* (**S2 Table**), 36 of the 94 genes (38%) regulated in *GRF9-IOE* are also similarily regulated in the *grf1*/*2*/*3* triple mutant (**S2 Table**) including *SAP12*, *ZAT6*, *ZAT10*, *ZAT11*, *ZAT12*, *WRKY40*, *WRKY48*, *WRKY54*, *ORE1* and several other genes that have a role in stress responses and ABA signalling [74–84]. Of note, the stress hormone ABA may function as an important factor in determining organ size [85–87], indicating that GRF9 plays a role in coordinating growth with stress responses as suggested for some other members of the GRF family [38,39]. Among the genes down-regulated in the *GRF9-IOE* line are *ARR6* (*TYPE-A ARABIDOPSIS RESPONSE REGULATOR6*) and *DOT1* (*DEFECTIVELY ORGANIZED TRIBUTARIES1*) which have been shown to positively function in leaf growth and development [88–91].

We also compared the list of differentially expressed genes in *GRF5ox* plants (producing bigger leaves [55]) and *grf7-1* (with smaller leaves [51]) with the genes regulated in *GRF9-IOE* seedlings after estradiol induction. We found only one gene, namely *WRKY54*, to be downregulated in *GRF5ox* but upregulated in *GRF9-IOE* plants, and 18 genes commonly upregulated in *grf7-1* and *GRF9-IOE* plants, of which several are stress related such as *WRKY40* and *ETHYLENE RESPONSE FACTOR* 105 (*ERF105*) transcription factors (**S2 Table**).

In conclusion, we have shown that GRF9 negatively regulates final leaf size by restricting cell proliferation predominantly in the incipient leaf primordium, and limiting cell proliferation in the young leaf, which may contribute to the positioning of the cell cycle arrest front. Another but here not investigated possibility is that GRF9 limits the number of leaf founder cells within the SAM. GRF9 acts in a transcriptional cascade together with *ORG3*, which it activates by directly binding to its promoter. In addition, *GRF9*, through the induction of ABA- and stress-responsive genes, may function in coordinating final organ size with stress responses.

## Materials and methods

### General

Standard molecular techniques were performed as described [92]. Chemicals and reagents were obtained from Sigma-Aldrich (Deisenhofen, Germany), Fluka (Buchs, Switzerland) or Merck (Darmstadt, Germany). Molecular biological reagents and kits were purchased from the suppliers indicated as well as from Roche (Mannheim, Germany) and Macherey-Nagel (Düren, Germany). Oligonucleotides were synthesized by MWG (Ebersberg, Germany) or GeneWorks (Adelaide, Australia). DNA sequencing was performed by MWG and GeneWorks. For sequence analysis, we employed the tools provided by the National Centre for Biotechnology Information (http://www.ncbi.nlm.nih.gov/) and the Arabidopsis Information Resource (TAIR; http://www.Arabidopsis.org/). Sequences of oligonucleotides used for making DNA constructs, for performing PCR, ChIP, and EMSA, and for genotyping are given in **S3 Table**. Data underlying figures and supplemental figures are given in **S4 Table**.

### Plant material and growth conditions

*Arabidopsis thaliana* (L.) Heynh., accession Col-0, was used as the wild-type control in all experiments. The *grf9-1* (SALK_140746c) and *grf9-2* (SAIL_324_G07) and *org3-1* (SALK_025676) and *org3-2* (SAIL_737_H11) mutants were obtained from the Nottingham Arabidopsis Stock Centre (NASC; http://arabidopsis.info). T-DNA insertions and genotypes were confirmed by PCR amplification using specific primers as described in the SIGnAL database (http://signal.salk.edu).

After imbibition, the seeds were stratified at 4°C for 3 days. The seeds were germinated at 22°C under a 16-h day (140 μmol m^-2^ s^-1^) / 8-h night regime. For histochemical GUS staining and hormone treatment assays, seeds were surface-sterilized for 15 min in 70% [v/v] ethanol and then in sterilisation solution (6% [w/v] sodium hypochlorite) for 10 min and thereafter washed three times with autoclaved ddH_2_O. After sterilization, the seeds were sown on half-strength MS medium (Murashige and Skoog, 1962), supplemented with 1% (w/v) sucrose and appropriate antibiotics, and solidified with 0.7% (w/v) phytoagar. Two-week-old Arabidopsis seedlings were carefully removed from plates and transplanted to soil (Einheitserde GS90; Gebrüder Patzer, Sinntal-Jossa, Germany) or, if necessary, directly subjected to various treatments. Unless otherwise indicated, Arabidopsis plants were grown in controlled conditions in a growth chamber with 16-h day length provided by fluorescent light at 80 or 120 μmol m^-2^ s^-1^, a day/night temperature of 20/16°C and relative humidity of 60/75%.

### Constructs

*GRF9-CELD*: The *GRF9* coding sequence (CDS) was PCR-amplified from Arabidopsis seedling cDNA using primers GRF9-CELD-fwd and GRF9-CELD-rev and inserted into pJET1.2 (Fermentas, Germany) from where it was then transferred via *Nhe*I and *Bam*HI sites into plasmid pTacLCELD6XHis [63] to create a GRF9-CELD in-frame fusion construct (pTacGRF9LCELD6XHis). *Pro*_*GRF9*_*-FLuc*: The ~1.5-kb *GRF9* promoter (upstream of the translation start codon) was amplified by PCR from Arabidopsis genomic DNA and inserted into pENTR/D-TOPO vector using the pENTR Directional TOPO Cloning Kit (Invitrogen, Germany). The sequence-verified promoter was then transferred to the p2GWL7.0 vector (Ghent University; http://gateway.psb.ugent.be/vector) harbouring the firefly luciferase (FLuc) coding region by LR recombination to generate *Pro*_*GRF9*_*-FLuc* (full-length *GRF9* promoter). *Pro*_*35S*_:*GRF9-GFP*: the *GRF9* CDS without its stop codon was amplified by PCR and inserted into the pENTR/D-TOPO vector using the pENTR Directional TOPO Cloning Kit (Invitrogen, Germany). The sequence-verified CDS was then transferred to the pK7FWG2 vector [93] by LR recombination. *Pro*_*GRF9*_:*GUS*: a ~1.5-kb fragment upstream of the *GRF9* translation initiation codon was amplified from genomic Arabidopsis Col-0 DNA by PCR using primers PGRF9-fwd and PGRF9-rev, inserted into pENTR/D-TOPO vector using the pENTR Directional TOPO Cloning Kit (Invitrogen, Germany). The sequence-verified CDS was then transferred to the pKGWFS7,0 vector [93]. *Pro*_*35S*_:*GRF9* and *Pro*_*35S*_:*ORG3*: the *GRF9* and *ORG3* CDSs, respectively, were amplified by PCR from Arabidopsis seedling cDNA, inserted individually into vector pJET1.2 (Fermentas), and then cloned via added *Pme*I and *Pac*I sites into a CaMV 35S-containing pGreen0229 vector (http://www.pgreen.ac.uk/). *GRF9-IOE*: the *GRF9* CDS was amplified by PCR from Arabidopsis seedlings cDNA using primers GRF9-IOE-fwd and GRF9-IOE-rev, inserted into pJET1.2 (Fermentas) and then cloned via *Xho*I and *Pac*I sites into pER8 vector [94] *Agrobacterium tumefaciens* strains GV3101 (pMP90) was used for *Arabidopsis thaliana* (Col-0) transformations.

### *In vitro* binding-site selection

GRF9-CELD fusion protein was prepared essentially as described by Xue (2005) [63], except that the following buffer for preparation and storage of GRF9-CELD protein was used: 10 mM sodium phosphate, pH7.2, 50 mM KCl, 0.5 mM DTT and 10 μM ZnCl_2_. The standard procedure for *in vitro* binding-site selection using Ni-NTA magnetic beads as an affinity matrix was used for selection of GRF9 binding sites [63], using a biotin-labelled double-stranded oligonucleotide containing a 30-nt random sequence [5’-CCAGGTGCGCTGGCGGACG(N30)GCTAGCCGATCGGAGCTCGG], except that MgCl_2_ in DNA-binding buffer and washing buffer was replaced with 10 μM and 1 μM ZnCl_2_, respectively. The GRF9-selected oligonucleotides after the sixth selection round were cloned and analysed for GRF9 binding activity. Positive clones were used for sequence analysis.

### GRF9 binding assay

The DNA-binding activity of GRF9-CELD was measured essentially as described previously [63,95] using streptavidin-coated 96-well plate and a binding buffer of 25 mM HEPES/KOH, pH 7.0, 50 mM KCl, 0.5 mM DTT and 10 μM ZnCl_2_, 0.15 μg μl^-1^ sheared herring sperm DNA, 0.3 mg ml^-1^ bovine serum albumin, 10% [w/v] glycerol and 0.025% [v/v] Nonidet P-40. 40,000 fluorescent units h^-1^ of the CELD activity of GRF9-CELD protein and 2 pmol of biotinylated probes were used per assay. The washing buffer contained 25 mM HEPES/KOH, pH 7.0, 50 mM KCl and 1 μM ZnCl_2_. The cellulase activity of GRF9-CELD protein bound to immobilised biotinylated probes was assayed by incubation in 100 μl of the CELD substrate solution (1 mM methylumbelliferyl β-D-cellobioside (MUC) in 50 mM Na-citrate buffer, pH 6.0) at 40°C for 3 h. A biotin-labelled double-stranded oligonucleotide without a GRF9 binding site was used as a control for background activity.

### Gene expression analysis by microarray hybridisation

Two micrograms of quality-checked total RNA obtained from either 2-week-old *GRF9-IOE* seedlings grown on MS medium (3 and 4 h after induction with 10 μM estradiol or 0.15% [v/v] ethanol for control) or detached mature leaves from 4-week-old soil-grown *GRF9-IOE* plants (6 h after 10 μM estradiol treatment or 0.15% [v/v] ethanol for control) were used for Affymetrix ATH1 micro-array hybridisations (one biological replicate each). Labelling, hybridisation, washing, staining, and scanning procedures were performed by ATLAS Biolabs (Berlin, Germany). Up-/down-regulated genes at each time point were obtained by calculating the ratio of the gene expression values in treatment versus control values. In addition, all time points were considered as three replicates and the differentially expressed genes were obtained using the limma package [96] in R (R Core Team, 2013). Expression data (GRF9-IOE-3h, GRF9-IOE-4h and GRF9-IOE-6h datasets) have been submitted to the NCBI Gene Expression Omnibus (GEO) repository (www.ncbi.nlm.nih.gov/geo/) and are available under accession number GSE98490.

### Quantitative RT-PCR

Quantitative RT-PCR was performed as previously described [97] using RNA extracted from 2-week-old *GRF9-IOE* seedlings grown on MS medium 1, 2, 3, 5 and 6 h after induction with 10 μM estradiol (or 0.15% [v/v] ethanol for control), or from 2-week-old *GRF9ox* and *grf9-1* seedlings grown on MS medium, by RNeasy Plant Mini Kit (Qiagen, Hilden, Germany). RNA was extracted from different tissues or whole seedlings (at least three biological replicates). Genomic DNA contamination was removed from RNA using Turbo DNA Free Kit (Ambion/Thermo Fisher Scientific, Darmstadt, Germany) and cDNA was synthesized by RevertAid First Strand cDNA Synthesis Kit using oligo(dT) primer (Thermo Fisher Scientific, Sankt-Leon Rot, Germany). Primers were designed using the QuantPrime tool ([98]; http://www.quantprime.de/). PCR reactions were run on an ABI PRISM 7900HT sequence detection system (Applied Biosystems), and the amplification products were visualized using SYBR Green (Applied Biosystems). Data were normalized against reference gene *ACTIN2* (*At3g18780*), and calculated using the ΔΔCt method [99]. Transcript levels are calculated as the difference between an arbitrary value of 40 and dCt, so that high 40-dCt value indicates high gene expression level. Relative expression shown in **Figs 1B and 3C** was calculated by dividing gene expression values of *grf9-2* or *GRF9ox1* by those of WT, respectively.

The level of mature *miR396* was determined as reported [100,101], with some modifications. In brief, RNA was extracted using TRIzol reagent (Invitrogen/Thermo Fisher Scientific, Sankt-Leon Rot, Germany) and genomic DNA contamination was removed from extracted RNA using Turbo DNA Free Kit (Ambion/Thermo Fisher Scientific, Darmstadt, Germany). cDNA was synthesized from 5 μg total RNA by SuperScript II reverse transcriptase (Invitrogen/Thermo Fisher Scientific, Sankt-Leon Rot, Germany) employing a mixture of oligo(dT) and stem-loop primers according to the instruction manual. PCR reactions were run on an ABI PRISM 7900HT sequence detection system (Applied Biosystems) using primers specific for *miR396*, *GRF9* and *ACTIN2* (**S3 Table**), and the amplification products were visualized using SYBR Green master mix (Applied Biosystems). Data normalization was done as explained in the previous paragraph.

### Semi-quantitative RT-PCR

Total RNA was extracted from *ORG3* transgenic and WT plants and genomic DNA contamination was removed using Turbo DNA Free Kit (Ambion/Thermo Fisher Scientific, Darmstadt, Germany). cDNA was synthesized by RevertAid First Strand cDNA Synthesis Kit (Thermo Fisher Scientific). RT-PCR was performed using *ORG3*-specific primers in a 25-μl reaction volume containing 1 μl cDNA, 2.5 μl 10 x DreamTag Green buffer (Thermo Fisher Scientific), 0.2 mM dNTPs, 1 μM each primer, and 1 U DreamTag DNA polymerase (Thermo Fisher Scientific). The PCR cycling program was as follows: initial denaturation of 1 min at 95°C, followed by 25 cycles of 95°C for 30 s, 57°C for 30 s and 72°C for 1 min. The final extension phase was 10 min at 72°C. *ACTIN2*-specific primers were used for control amplifications.

### Microscopic analyses

Whole leaves and leaf cells were observed with a stereoscopic microscope (Lumar, Carl Zeiss, Jena, Germany) and a Nomarski differential interference contrast microscope (BX51, Olympus, Tokyo, Japan), respectively. For histological analysis of cells, the first set of leaves from 20-day-old plants were collected. The leaves were fixed in FAA (formalin: acetic acid: ethanol, 1: 1: 18) and cleared using chloral hydrate solution (chloral hydrate 100 g, glycerol 10 g, water 25 ml), as described before [102]. Whole leaves and cells were observed as previously described [103]. Means ± SD from at least eight individual plants are given in the figures. To calculate the total cell number in leaves, we measured cell density of observed images of cells, and multiplied the cell density by the area of the same leaf. Kinetic analyses were performed as described [11].

Size of cells in leaf primodia and cell number in the layer 1 (L1) of the SAM were analysed in Fiji (https://fiji.sc/) using images generated with a Nikon eclipse E600 microscope. Means ± SEM from at least eight individual plants are given in the figures.

### Visualization of cell proliferating with 5-ethynyl-2'-deoxyuridine (EdU)

To detect proliferating cells, we used the EdU assay, which stains S-phase cells [56]. Five-day-old-seedlings were incubated with 10 μm EdU (Invitrogen, cat no: A10044; dissolved in water) for 4 h under illumination in a culture chamber. After incubation, samples were fixed in 90% [v/v] acetone for 10 min, washed twice with phosphate buffered saline (PBS) buffer (pH 7.0). Subsequently, the buffer was replaced with FAA and incubated under vacuum for 2 h. Fixed seedlings were washed twice with 0.5% [v/v] Triton X-100 in PBS for 5 min each, then washed again with PBS for 5 min. Next, samples were incubated in EdU detection cocktail (10 μm Alexa 488, Invitrogen; 100 mM Tris-HCl, pH 8.5, 1 mM CuSO_4_, 100 mM ascorbate) for 30 min. Finally, samples were rinsed three times with PBS for 20 min each, then transferred onto microscopy slides and covered with a chloral hydrate solution to make the samples transparent. Observations were done using a confocal laser scanning microscope (LSM 710; Zeiss, Jena, Germany). EdU signals were scanned and calculated by ImageJ (http://imagej.nih.gov/ij/).

### Tissue embedding, sectioning and RNA *in situ* hybridization

Meristems of 2-day-old WT and *GRF9* transgenic plants grown in long days (16 h light/8 h dark) were harvested, fixed, embedded into wax using an automated tissue processor (ASP200S; Leica, Wetzlar, Deutschland) and embedding system (HistoCore Arcadia; Leica). Sections of 8 μm thickness were prepared using a rotary microtome (RM2255; Leica). Briefly, RNA *in situ* hybridization was carried out by dewaxing slides containing sections in Histoclear solution (Biozym Scientific, Hessisch Oldendorf, Germany) and processed through ethanol series. Then, the slides were incubated in Proteinase K (Roche, Mannheim, Germany) and dehydrated by processing through an ethanol series. Further, *HISTONE4 (H4*) or *CYCB1;1* antisense probes mixed with hybridisation buffer were applied to the slides and hybridized overnight. The *H4* and *CYCB1;1* probes were amplified and cloned into pGEM-T Easy Vector (Promega, Madison, Wisconsin, USA) and synthesized with the DIG RNA Labeling Kit (Roche). After the hybridization overnight, slides were washed out and incubated with 1% blocking reagent (Roche) in 1 × TBS /0.1% Triton X-100. For immunological detection, the Anti-DIG antibody (Roche) solution diluted 1:1,250 in blocking reagent was applied to the slides. Then, the slides were washed and for the colorimetric detection, the NBT/BCIP stock solution (Roche) diluted 1:50 in 10% polyvinyl alcohol (PVA) in TNM-50 was applied to the slides. The slides were incubated overnight and imaged with a Nikon eclipse E600 microscope (Nikon, Dűsseldorf, Germany) using NIS-Elements BR 4.51.00 software (Nikon). The figure panel was generated in Adobe Photoshop CS5 (Adobe Systems, San Jose, USA).

For toluidine blue staining slides were dewaxed by incubating in Histoclear and an ethanol series: 100% EtOH for 2 min, 100% EtOH for 2 min, 95% EtOH for 1 min, 90% EtOH for 1 min, 80% EtOH for 1 min, 60% EtOH + 0.75% of NaCl for 1 min, 30% EtOH + 0.75% of NaCl for 1 min, 0.75% NaCl for 1 min, and PBS for 1 min. The slides were shortly left to dry at 42°C and then incubated in 0.01% toluidine blue/sodium borate solution for 2 min, briefly washed with water and 80% EtOH. The sections were imaged as described above.

### Rosette growth analysis using a phenotyping platform

To perform whole-rosette phenotyping (**S4A and S4B Fig**), we used a growth phenotyping pipeline previously established [67]. To this end, Col-0, *grf9-2* and *GRF9ox-1* were sown in 5-cm-diameter pots in a 54-pot tray (QuickPot 54R, HerkuPlast-Kubern, Ering am Inn, Germany; http://www.herkuplast.com). Plants were grown in growth cabinets with a tightly controlled environment (Percival Scientific Inc., Perry, IO, USA http://www.percival-scientific.com) at 22°C and 70% relative humidity during the day, 18°C and 80% humidity at night, at a 12 h day: 12 h night cycle (equal day), or an 8 h day: 16 h night cycle (short day). Homogenized stratification was maximized by keeping the seeds for uniform germination at 6°C and 80% humidity for the first 7 nights (day condition was the same as indicated before). Developmental stages were defined as reported [67]. Images were captured by a robot arm holding a camera of the Scanalyzer HTS instrument (LemnaTec, Wuerselen, Germany, http://www.lemnatec.com). Image analysis was performed using LemnaGrid (provided with the image capturing system) and growth analysis was done using the growth phenotyping pipeline [67].

### Plant phenotyping

Organ and cell sizes were determined as described [104,105].

### GUS assays

Histochemical GUS assays were performed as explained previously [95,106].

### Electrophoretic mobility shift assay (EMSA)

EMSA was performed with GRF9-CELD fusion protein as described previously [107,108] using 5'-DY682-labelled oligonucleotides harbouring the GRF9 BS (5'-CTGACA-3'). Oligonucleotides were purchased from MWG Eurofins Genomics (Ebersberg, Germany).

### Transactivation assay

Isolation and transformation of Arabidopsis mesophyll cell protoplasts were done based on the tape-sandwich method [109]. Assays were performed as described [107], using *Pro*_*35S*_:*GRF9* effector plasmid. Luciferase activity was determined using the Dual-Luciferase Reporter Assay System (Promega, Germany) and a GloMax 96 microplate luminometer (Promega, Germany). All tests were performed in three biological replications with five technical replications per assay.

### ChIP-qPCR

Five-day-old Arabidopsis seedlings expressing GFP-tagged GRF9 protein from the CaMV 35S promoter (*Pro*_*35S*_:*GRF9-GFP*) were used for ChIP-qPCR. ChIP was done as reported [110]. μMACS GFP Isolation Kit (Miltenyi, Germany) was used for immunopurification of GRF9-GFP-DNA complex. qPCR was performed using primers flanking the GRF9 binding site of the *ORG3* promoter. Primers detecting enrichment of a promoter region lacking GRF9 binding site (*At2g22180*) was used for negative control. The relative enrichment was calculated by the comparative cycle threshold method [99]. The amounts of immunoprecipitated DNA were normalized to the input fraction. To calculate fold enrichment, normalized ChIP signals were compared between *Pro*_*35S*_:*GRF9-GFP* and wild-type plants as control [107], where the ChIP signal is given as the fold increase in signal relative to the background signal.

### AGI codes

AT2G45480, *GRF9*; AT3G56980, *ORG3*; AT2G28740, *HISTONE4;* AT4G37490, *CYCB1;1*. Other AGI codes are given in **S1–S4 Tables**.

## Supporting information

S1 Table Genes affected in *GRF9-IOE* lines after EST induction and comparison with other *GRF9*-modified plants. (XLSX)

S2 Table GRF9-responsive genes in *GRF9-IOE* seedlings compared to differentially expressed genes in other publically available AtGRF transcriptomes. (XLSX)

S3 Table List of primers used in this study. (XLS)

S4 Table Data corresponding to Figures and Supplemental Figures. (XLSX)

S1 Fig Analysis of *GRF9* promoter-driven reporter activity in *Pro*_*GRF9*_:*GUS* lines. (A)–(D) Histochemical GUS staining of *GRF9* expression pattern in leaves of 4-, 6-, 8-, and 12-day-old seedlings, respectively. (E) and (F) Leaves of 3-week-old plants. Note the expression of *GRF9* in the cell proliferation zone of very young leaves (B, C) and the vascular tissue of older leaves (D—F). (G) and (H) Main and lateral roots. (I)–(L) Flowers at different developmental stages. Note, that younger flowers show stronger GUS activity. (M)–(P) Siliques at different developmental stages.
(PDF)

S2 Fig Expression of *GRF9* determined by qRT-PCR. (A) *GRF9* expression in different tissues of 40-day-old WT plants. (B) *GRF9* expression in 2-week-old WT seedlings treated with different concentrations of auxin (in the form of 2,4-D) or cytokinin (in the form of zeatin). (C) Histochemical GUS staining of *GRF9* expression pattern in young Arabidopsis Col-0 seedlings treated with auxin (in the form of 2,4-D) or cytokinin (in the form of zeatin). Values in panels A and B represent the means ± SD of three technical replicates from two biological replicates.
(PDF)

S3 Fig Genotyping of *grf9-1* and *grf9-2* mutants. (A) *grf9-1* (SALK_140746c) and (B) *grf9-2* (SAIL_324_G07). (a) Right gene-specific primer and T-DNA left border primer, and (b) left and right gene-specific primers for genotyping (designed by http://signal.salk.edu/tdnaprimers.2.html). M, DNA size marker. Primer sequences are given in **S3 Table**.
(PDF)

S4 Fig Rosette growth of *GRF9* transgenic lines under different light regimes. Rosette phenotype of *grf9-2* and *GRF9ox1* in comparison to WT plants in (A) short day (8 h light / 16 h dark) and (B) equal day (12 h light / 12 h dark) conditions, determined using a LemnaTec phenotyping platform [67]. Note the more pronounced phenotype of the *grf9* mutant in short-day condition. (C) Rosette area determined at 21 days after sowing (DAS) for short-day-grown plants, and at 23 DAS for plants grown in equal day/night length. Values represent means ± SD of at least 50 plants each. Asterisks indicate significant difference from the WT (Student's *t*-test; *p* < 0.05).
(PDF)

S5 Fig RNA *in situ* hybridization using the *CYCLIN B1;1* (*CYCB1;1*) probe. *In situ* hybridization was done on longitudinal sections of the shoot apical meristem with leaf primordia of WT and *grf9-2* plants (Scale bar 100 μm).
(PDF)

S6 Fig Petal phenotype of *grf9* and *GRF9ox* plants. (A) Mature flowers and petals of WT, *grf9-1*, *grf9-2* and *GRF9ox1* plants. (B) Petal size and (C) petal cell area. Data represent means ± SD from at least 32 petals (i.e., 4 petals from at least 8 plants). Asterisks indicate a significant difference from the WT (Student's *t*-test; *p* < 0.05). Scale bars = 1 mm (panel A, top) and 0.5 mm (panel A, bottom).
(PDF)

S7 Fig Base substitution analysis of the GRF9 binding site. The experiment was performed to define the DNA-binding sequence specificity of GRF9 by base substitution mutagenesis. Biotin-labelled double-stranded oligonucleotides were used. Bases that were substituted are shown in bold and as lower-case letters. The values for GRF9 binding activity are shown on the right and are means ± SD of three independent assays, relative to the binding activity of GRFE1 (1,778 fluorescence units per h produced by the CELD activity of GRF9-CELD fusion protein). The core GRF9 binding sequence defined by this analysis is CTGACA.
(PDF)

S8 Fig Expression of 23 GRF9 early responding genes in different *GRF9*-modified lines. Gene expression as determined by Affymetrix ATH1 microarray hybridizations (first two columns) or qRT-PCR (other columns). RNA for expression analysis was obtained from 2-week-old *GRF9-IOE* seedlings grown on MS medium and induced with 10 μM estradiol for the indicated time points (0.15% [v/v] ethanol as control), or from 2-week-old *GRF9ox* and *grf9-1* seedlings grown on MS medium (WT as control). Values represent the means of replicates obtained from three sets of seedlings (except for the microarray data where each value represents one replicate).
(PDF)

S9 Fig Genotyping and expression analysis in *GRF9*- and *ORG3-*modified lines. Genotyping of (A) *org3-1* (SALK_025676) and (B) *org3-2* (SAIL_737_H11) mutants. (a) Right gene-specific primer and T-DNA left boarder primer, and (b) left and right gene-specific primers for genotyping (designed by http://signal.salk.edu/tdnaprimers.2.html). M, DNA size marker. Primer sequences are given in **S3 Table.** (C) Semi-quantitative RT-PCR using *ORG3*-specific primers performed on total RNA isolated from 1-week-old *org3-1*, *org3-2*, WT, *ORG3ox1* and *ORG3ox2* seedlings. *ACTIN2* was used as a control. (D) Expression of *ORG3* measured by qRT-PCR in *org3* knockout and *ORG3ox* plants. (E) Expression of *GRF9* and *ORG3* measured by qRT-PCR in *grf9-2 org3-1* (lines 3 and 7) and *GRF9ox-1 org3-1* (lines 33 and 34) double mutants. Values in panels D and E represent the means of three technical replicates ± SD. (F) DNA genotyping results of double mutant lines using (a) right gene-specific primer and T-DNA left boarder primer, (b) left and right gene-specific primers for genotyping (designed by http://signal.salk.edu/tdnaprimers.2.html), and (c) 35S-up and reverse *GRF9*-*IOE* specific primers. Genes tested by the chosen primer combinations are underlined. M, DNA size marker.
(PDF)

S10 Fig Petal phenotype of the *org3-1* mutant. (A) Mature petals of WT and *org3-1* plants. (B) Petal size and (C) petal cell area. Data represent means ± SD from at least 32 petals (i.e., 4 petals from at least 8 plants). The asterisk indicates a significant difference from WT (Student's *t*-test; *p* < 0.05). Bar = 0.5 mm.
(PDF)
