# Supplementary material for: GROWTH-REGULATING FACTOR 9 negatively regulates arabidopsis leaf growth by controlling ORG3 and restricting cell proliferation in leaf primordia
Source: PLoS Genet. 2018 Jul 9;14(7):e1007484. doi: 10.1371/journal.pgen.1007484 (PMC6053248; doi:10.1371/journal.pgen.1007484)
Supplement: S1 Fig — (A)–(D) Histochemical GUS staining of GRF9 expression pattern in leaves of 4-, 6-, 8-, and 12-day-old seedlings, respectively. (E) and (F) Leaves of 3-week-old plants. Note the expression of GRF9 in the cell proliferation zone of very young leaves (B, C) and the vascular tissue of older leaves (D—F). (G) and (H) Main and lateral roots. (I)–(L) Flowers at different developmental stages. Note, that younger flowers show stronger GUS activity. (M)–(P) Siliques at different developmental stages. (PDF) [file pgen.1007484.s005.pdf]

S1 Fig

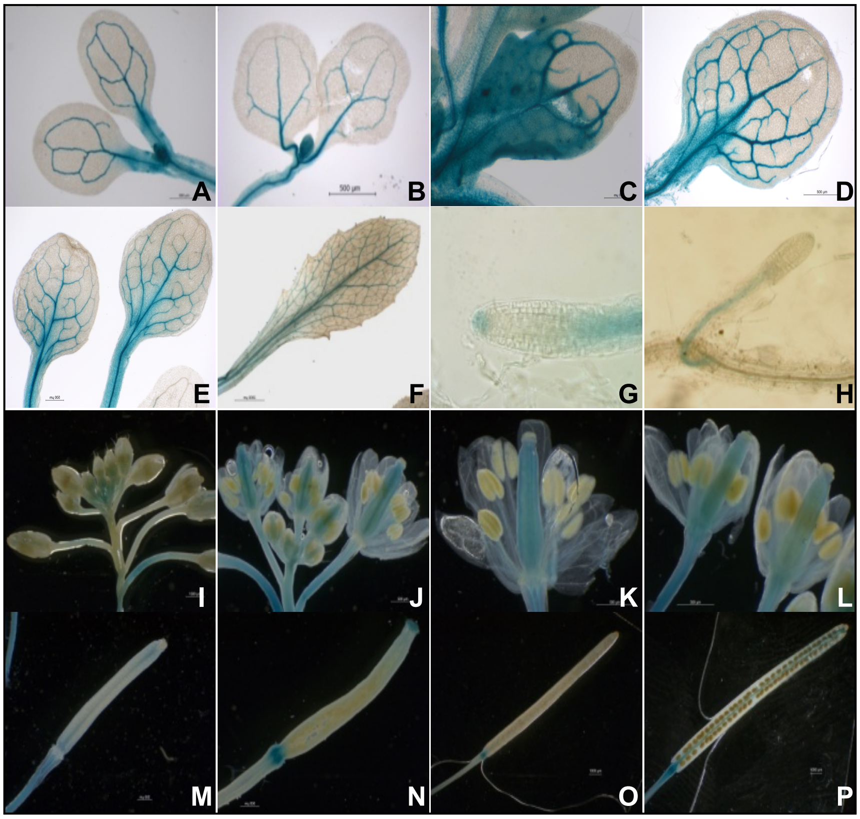

S1 Fig. Analysis of *GRF9* promoter-driven reporter activity in *Pro$_{GRF9}$:GUS* lines. (A) – (D) Histochemical GUS staining of *GRF9* expression pattern in leaves of 4-, 6-, 8-, and 12-day-old seedlings, respectively. (E) and (F) Leaves of 3-week-old plants. Note the expression of *GRF9* in the cell proliferation zone of very young leaves (B, C) and the vascular tissue of older leaves (D - F). (G) and (H) Main and lateral roots. (I) – (L) Flowers at different developmental stages. Note, that younger flowers show stronger GUS activity. (M) – (P) Siliques at different developmental stages.
