# Supplementary material for: GROWTH-REGULATING FACTOR 9 negatively regulates arabidopsis leaf growth by controlling ORG3 and restricting cell proliferation in leaf primordia
Source: PLoS Genet. 2018 Jul 9;14(7):e1007484. doi: 10.1371/journal.pgen.1007484 (PMC6053248; doi:10.1371/journal.pgen.1007484)
Supplement: S2 Fig — (A) GRF9 expression in different tissues of 40-day-old WT plants. (B) GRF9 expression in 2-week-old WT seedlings treated with different concentrations of auxin (in the form of 2,4-D) or cytokinin (in the form of zeatin). (C) Histochemical GUS staining of GRF9 expression pattern in young Arabidopsis Col-0 seedlings treated with auxin (in the form of 2,4-D) or cytokinin (in the form of zeatin). Values in panels A and B represent the means ± SD of three technical replicates from two biological replicates. (PDF) [file pgen.1007484.s006.pdf]

S2 Fig

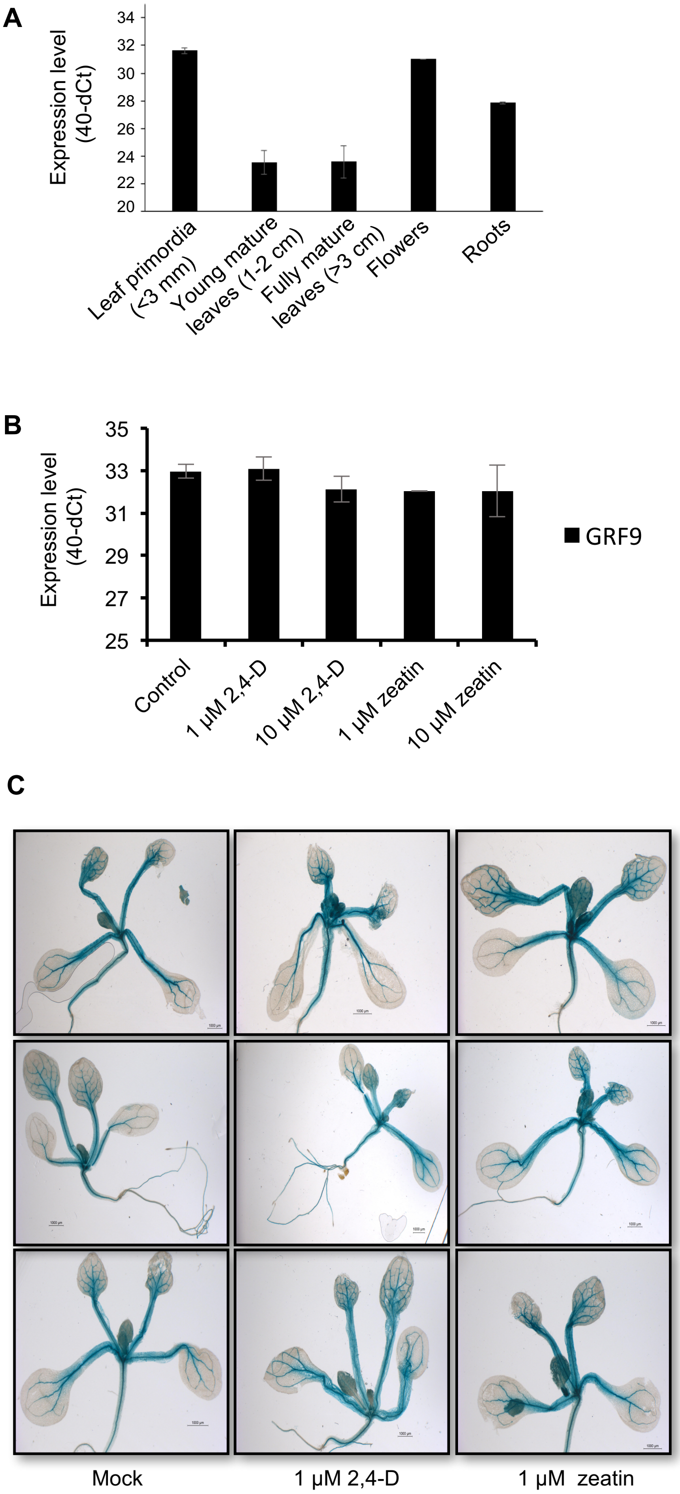

S2 Fig. Expression of *GRF9* determined by qRT-PCR. (A) *GRF9* expression in different tissues of 40-day-old WT plants. (B) *GRF9* expression in 2-week-old WT seedlings treated with different concentrations of auxin (in the form of 2,4-D) or cytokinin (in the form of zeatin). (C) Histochemical GUS staining of *GRF9* expression pattern in young Arabidopsis Col-0 seedlings treated with auxin (in the form of 2,4-D) or cytokinin (in the form of zeatin). Values in panels A and B represent the means ± SD of three technical replicates from two biological replicates.
