# Supplementary material for: GROWTH-REGULATING FACTOR 9 negatively regulates arabidopsis leaf growth by controlling ORG3 and restricting cell proliferation in leaf primordia
Source: PLoS Genet. 2018 Jul 9;14(7):e1007484. doi: 10.1371/journal.pgen.1007484 (PMC6053248; doi:10.1371/journal.pgen.1007484)
Supplement: S3 Fig — (A) grf9-1 (SALK_140746c) and (B) grf9-2 (SAIL_324_G07). (a) Right gene-specific primer and T-DNA left border primer, and (b) left and right gene-specific primers for genotyping (designed by http://signal.salk.edu/tdnaprimers.2.html). M, DNA size marker. Primer sequences are given in S3 Table. (PDF) [file pgen.1007484.s007.pdf]

S3 Fig

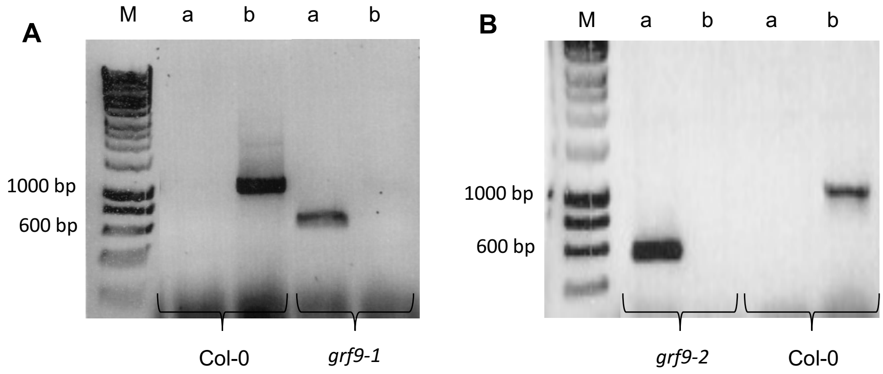

S3 Fig. Genotyping of *grf9-1* and *grf9-2* mutants. (A) *grf9-1* (SALK_140746c) and (B) *grf9-2* (SAIL_324_G07). (a) Right gene-specific primer and T-DNA left border primer, and (b) left and right gene-specific primers for genotyping (designed by http://signal.salk.edu/tdnaprimers.2.html). M, DNA size marker. Primer sequences are given in **S3 Table**.
