# Supplementary material for: GROWTH-REGULATING FACTOR 9 negatively regulates arabidopsis leaf growth by controlling ORG3 and restricting cell proliferation in leaf primordia
Source: PLoS Genet. 2018 Jul 9;14(7):e1007484. doi: 10.1371/journal.pgen.1007484 (PMC6053248; doi:10.1371/journal.pgen.1007484)
Supplement: S4 Fig — Rosette phenotype of grf9-2 and GRF9ox1 in comparison to WT plants in (A) short day (8 h light / 16 h dark) and (B) equal day (12 h light / 12 h dark) conditions, determined using a LemnaTec phenotyping platform [67]. Note the more pronounced phenotype of the grf9 mutant in short-day condition. (C) Rosette area determined at 21 days after sowing (DAS) for short-day-grown plants, and at 23 DAS for plants grown in equal day/night length. Values represent means ± SD of at least 50 plants each. Asterisks indicate significant difference from the WT (Student's t-test; p < 0.05). (PDF) [file pgen.1007484.s008.pdf]

S4 Fig

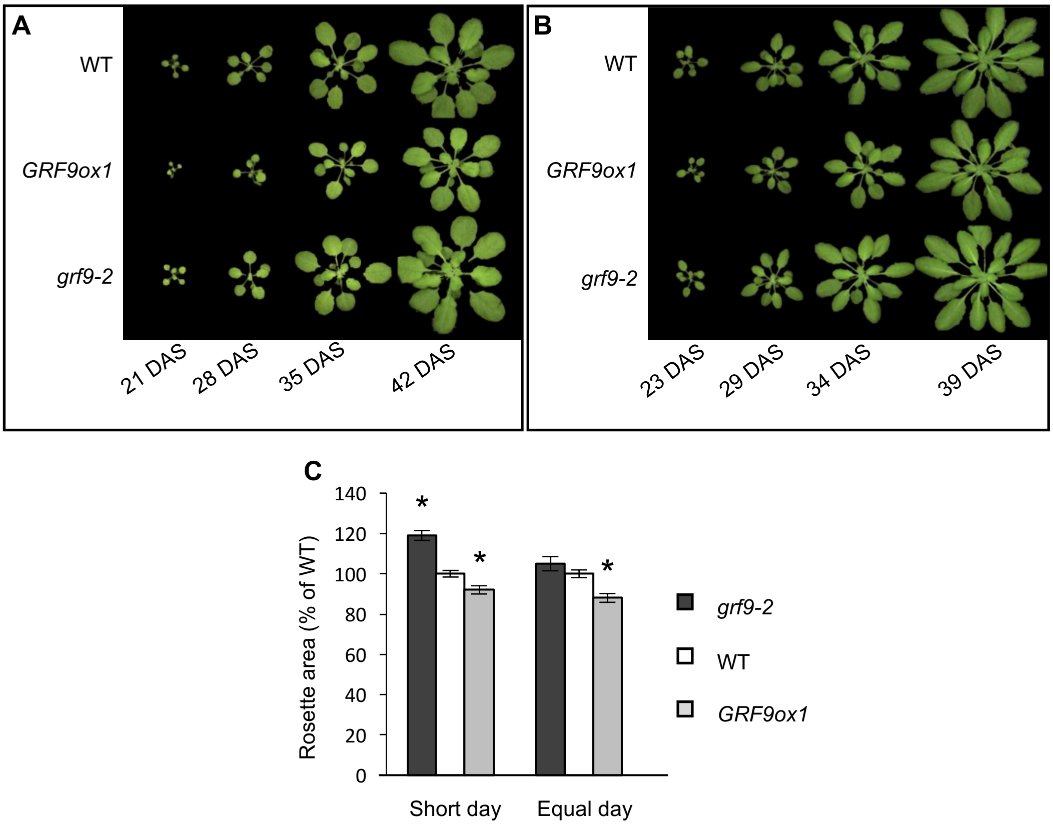

S4 Fig. Rosette growth of *GRF9* transgenic lines under different light regimes. Rosette phenotype of *grf9-2* and *GRF9ox1* in comparison to WT plants in (A) short day (8 h light / 16 h dark) and (B) equal day (12 h light / 12 h dark) conditions, determined using a LemnaTec phenotyping platform [67]. Note the more pronounced phenotype of the *grf9* mutant in short-day condition. (C) Rosette area determined at 21 days after sowing (DAS) for short-day-grown plants, and at 23 DAS for plants grown in equal day/night length. Values represent means ± SD of at least 50 plants each. Asterisks indicate significant difference from the WT (Student´s *t*-test; $p < 0.05$).
