# Supplementary material for: GROWTH-REGULATING FACTOR 9 negatively regulates arabidopsis leaf growth by controlling ORG3 and restricting cell proliferation in leaf primordia
Source: PLoS Genet. 2018 Jul 9;14(7):e1007484. doi: 10.1371/journal.pgen.1007484 (PMC6053248; doi:10.1371/journal.pgen.1007484)
Supplement: S5 Fig — In situ hybridization was done on longitudinal sections of the shoot apical meristem with leaf primordia of WT and grf9-2 plants (Scale bar 100 μm). (PDF) [file pgen.1007484.s009.pdf]

S5 Fig

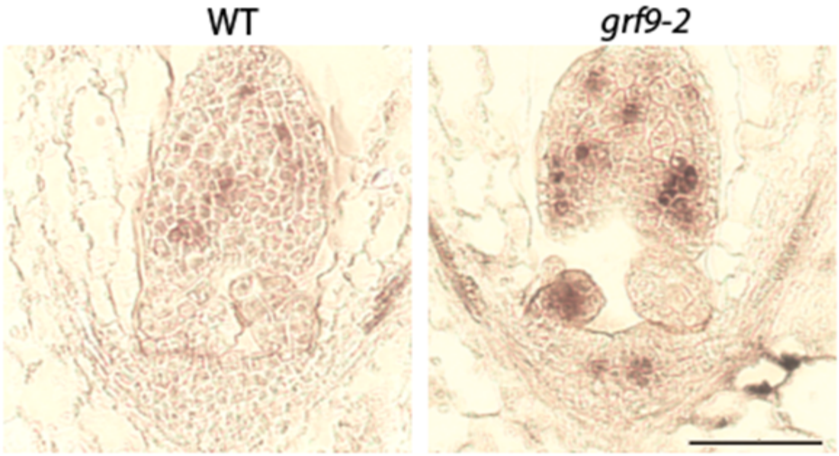

S5 Fig. RNA *in situ* hybridization using the *CYCLIN B1;1* (*CYCB1;1*) probe. *In situ* hybridization was done on longitudinal sections of the shoot apical meristem with leaf primordia of WT and *grf9-2* plants (Scale bar 100 µm).
