# Supplementary material for: GROWTH-REGULATING FACTOR 9 negatively regulates arabidopsis leaf growth by controlling ORG3 and restricting cell proliferation in leaf primordia
Source: PLoS Genet. 2018 Jul 9;14(7):e1007484. doi: 10.1371/journal.pgen.1007484 (PMC6053248; doi:10.1371/journal.pgen.1007484)
Supplement: S6 Fig — (A) Mature flowers and petals of WT, grf9-1, grf9-2 and GRF9ox1 plants. (B) Petal size and (C) petal cell area. Data represent means ± SD from at least 32 petals (i.e., 4 petals from at least 8 plants). Asterisks indicate a significant difference from the WT (Student's t-test; p < 0.05). Scale bars = 1 mm (panel A, top) and 0.5 mm (panel A, bottom). (PDF) [file pgen.1007484.s010.pdf]

S6 Fig

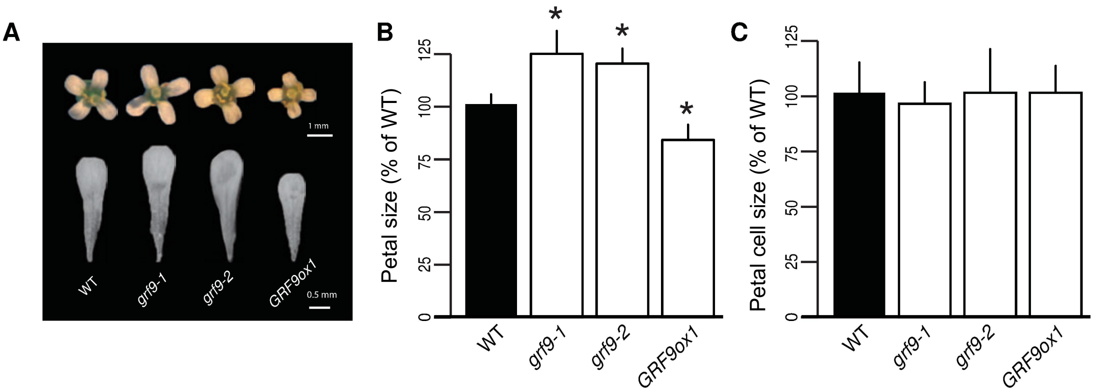

S6 Fig. Petal phenotype of *grf9* and *GRF9ox* plants. (A) Mature flowers and petals of WT, *grf9-1*, *grf9-2* and *GRF9ox1* plants. (B) Petal size and (C) petal cell area. Data represent means ± SD from at least 32 petals (i.e., 4 petals from at least 8 plants). Asterisks indicate a significant difference from the WT (Student´s *t*-test; $p < 0.05$). Scale bars = 1 mm (panel A, top) and 0.5 mm (panel A, bottom).
