# Supplementary material for: GROWTH-REGULATING FACTOR 9 negatively regulates arabidopsis leaf growth by controlling ORG3 and restricting cell proliferation in leaf primordia
Source: PLoS Genet. 2018 Jul 9;14(7):e1007484. doi: 10.1371/journal.pgen.1007484 (PMC6053248; doi:10.1371/journal.pgen.1007484)
Supplement: S7 Fig — The experiment was performed to define the DNA-binding sequence specificity of GRF9 by base substitution mutagenesis. Biotin-labelled double-stranded oligonucleotides were used. Bases that were substituted are shown in bold and as lower-case letters. The values for GRF9 binding activity are shown on the right and are means ± SD of three independent assays, relative to the binding activity of GRFE1 (1,778 fluorescence units per h produced by the CELD activity of GRF9-CELD fusion protein). The core GRF9 binding sequence defined by this analysis is CTGACA. (PDF) [file pgen.1007484.s011.pdf]

S7 Fig

Probe name	Base substitution analysis	Relative GRF9 binding activity
GRFE1	CCGGTACTGGTCTGACATACCAGAACTTCA	1.00 ± 0.05
GRFE15	AGGTAAGTTACTGACACACTCCATTCTTGG	0.80 ± 0.03
GRFE1m1	CCGGTACTGG**a**CTGACA**c**ACCAGAACTTCA	0.79 ± 0.03
GRFE1m2	CCGGTACTGG**c**CTGACA**c**ACCAGAACTTCA	0.43 ± 0.04
GRFE1m3	CCGGTACTGGTCTGACA**a**ACCAGAACTTCA	0.89 ± 0.08
GRFE1m4	CCGGTACTGGT**g**TGACATACCAGAACTTCA	0
GRFE1m5	CCGGTACTGGTCTGAC**t**TACCAGAACTTCA	0
GRFE1m6	CCGGTACTGGTC**g**GACATACCAGAACTTCA	0
GRFE1m7	CCGGTACTGGTCTGA**g**ATACCAGAACTTCA	0

S7 Fig. Base substitution analysis of the GRF9 binding site. The experiment was performed to define the DNA-binding sequence specificity of GRF9 by base substitution mutagenesis. Biotin-labelled double-stranded oligonucleotides were used. Bases that were substituted are shown in bold and as lower-case letters. The values for GRF9 binding activity are shown on the right and are means ± SD of three independent assays, relative to the binding activity of GRFE1 (1,778 fluorescence units per h produced by the CELD activity of GRF9-CELD fusion protein). The core GRF9 binding sequence defined by this analysis is CTGACA.
