# Supplementary material for: GROWTH-REGULATING FACTOR 9 negatively regulates arabidopsis leaf growth by controlling ORG3 and restricting cell proliferation in leaf primordia
Source: PLoS Genet. 2018 Jul 9;14(7):e1007484. doi: 10.1371/journal.pgen.1007484 (PMC6053248; doi:10.1371/journal.pgen.1007484)
Supplement: S8 Fig — Gene expression as determined by Affymetrix ATH1 microarray hybridizations (first two columns) or qRT-PCR (other columns). RNA for expression analysis was obtained from 2-week-old GRF9-IOE seedlings grown on MS medium and induced with 10 μM estradiol for the indicated time points (0.15% [v/v] ethanol as control), or from 2-week-old GRF9ox and grf9-1 seedlings grown on MS medium (WT as control). Values represent the means of replicates obtained from three sets of seedlings (except for the microarray data where each value represents one replicate). (PDF) [file pgen.1007484.s012.pdf]

S8 Fig

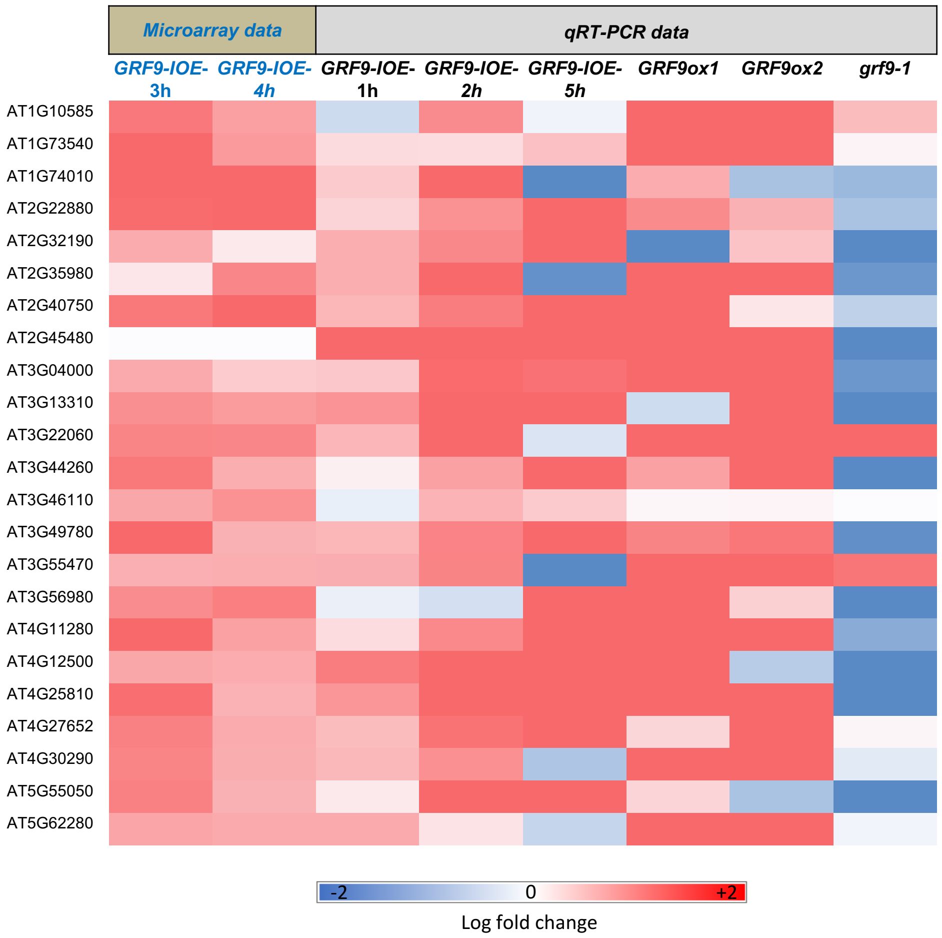

S8 Fig. Expression of 23 GRF9 early responding genes in different *GRF9*-modified lines. Gene expression as determined by Affymetrix ATH1 microarray hybridizations (first two columns) or qRT-PCR (other columns). RNA for expression analysis was obtained from 2-week-old *GRF9-IOE* seedlings grown on MS medium and induced with 10 µM estradiol for the indicated time points (0.15% [v/v] ethanol as control), or from 2-week-old *GRF9ox* and *grf9-1* seedlings grown on MS medium (WT as control). Values represent the means of replicates obtained from three sets of seedlings (except for the microarray data where each value represents one replicate).
