# Supplementary material for: GROWTH-REGULATING FACTOR 9 negatively regulates arabidopsis leaf growth by controlling ORG3 and restricting cell proliferation in leaf primordia
Source: PLoS Genet. 2018 Jul 9;14(7):e1007484. doi: 10.1371/journal.pgen.1007484 (PMC6053248; doi:10.1371/journal.pgen.1007484)
Supplement: S9 Fig — Genotyping of (A) org3-1 (SALK_025676) and (B) org3-2 (SAIL_737_H11) mutants. (a) Right gene-specific primer and T-DNA left boarder primer, and (b) left and right gene-specific primers for genotyping (designed by http://signal.salk.edu/tdnaprimers.2.html). M, DNA size marker. Primer sequences are given in S3 Table. (C) Semi-quantitative RT-PCR using ORG3-specific primers performed on total RNA isolated from 1-week-old org3-1, org3-2, WT, ORG3ox1 and ORG3ox2 seedlings. ACTIN2 was used as a control. (D) Expression of ORG3 measured by qRT-PCR in org3 knockout and ORG3ox plants. (E) Expression of GRF9 and ORG3 measured by qRT-PCR in grf9-2 org3-1 (lines 3 and 7) and GRF9ox-1 org3-1 (lines 33 and 34) double mutants. Values in panels D and E represent the means of three technical replicates ± SD. (F) DNA genotyping results of double mutant lines using (a) right gene-specific primer and T-DNA left boarder primer, (b) left and right gene-specific primers for genotyping (designed by http://signal.salk.edu/tdnaprimers.2.html), and (c) 35S-up and reverse GRF9-IOE specific primers. Genes tested by the chosen primer combinations are underlined. M, DNA size marker. (PDF) [file pgen.1007484.s013.pdf]

S9 Fig

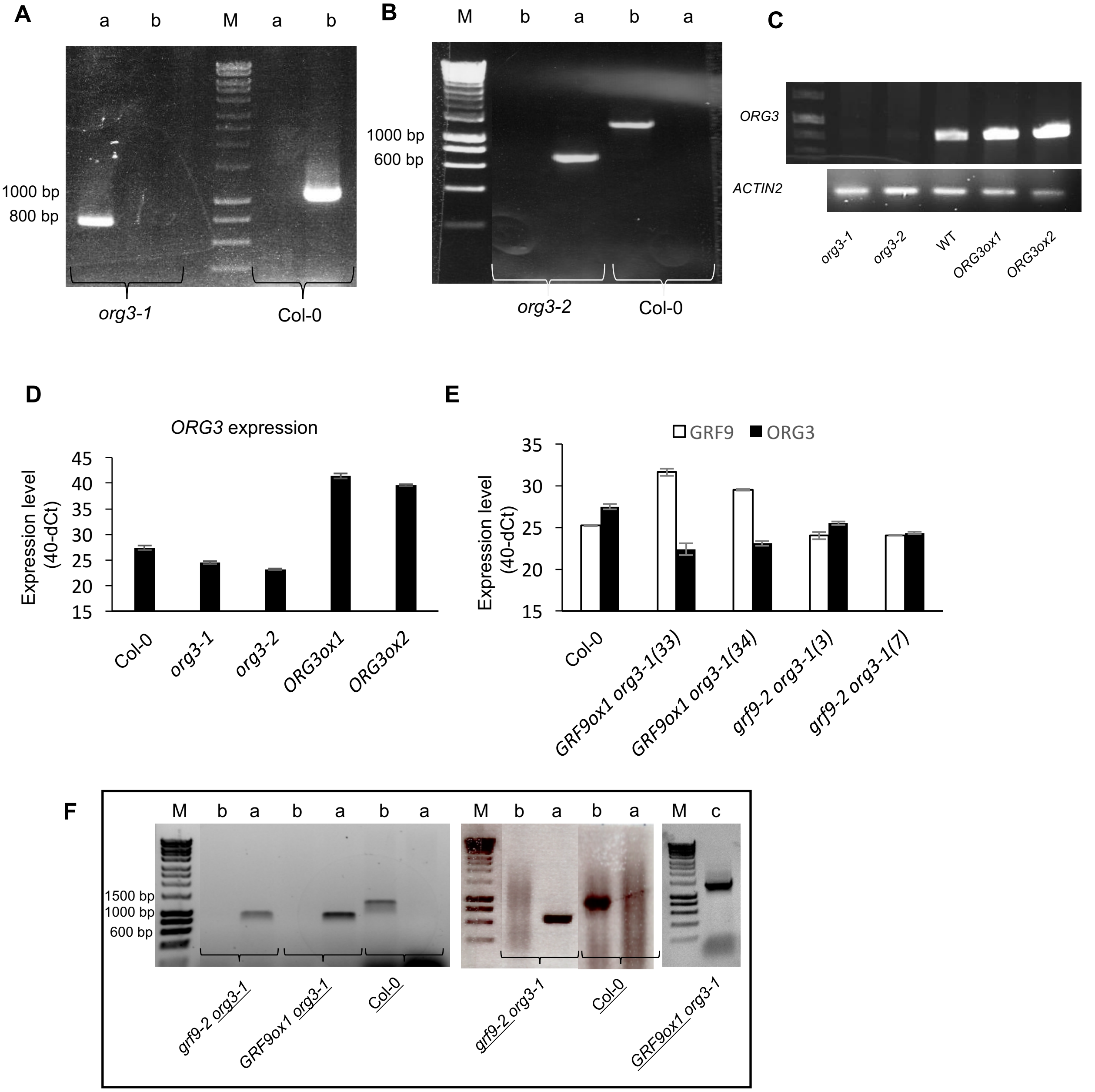

S9 Fig. Genotyping and expression analysis in *GRF9*- and *ORG3*-modified lines. Genotyping of (A) *org3-1* (SALK_025676) and (B) *org3-2* (SAIL_737_H11) mutants. (a) Right gene-specific primer and T-DNA left boarder primer, and (b) left and right gene-specific primers for genotyping (designed by http://signal.salk.edu/tdnaprimers.2.html). M, DNA size marker. Primer sequences are given in **S3 Table.** (C) Semi-quantitative RT-PCR using *ORG3*-specific primers performed on total RNA isolated from 1-week-old *org3-1*, *org3-2*, WT, *ORG3ox1* and *ORG3ox2* seedlings. *ACTIN2* was used as a control. (D) Expression of *ORG3* measured by qRT-PCR in *org3* knockout and *ORG3ox* plants. (E) Expression of *GRF9* and *ORG3* measured by qRT-PCR in *grf9-2 org3-1* (lines 3 and 7) and *GRF9ox-1 org3-1* (lines 33 and 34) double mutants. Values in panels D and E represent the means of three technical replicates ± SD. (F) DNA genotyping results of double mutant lines using (a) right gene-specific primer and T-DNA left boarder primer, (b) left and right gene-specific primers for genotyping (designed by http://signal.salk.edu/tdnaprimers.2.html), and (c) 35S-up and reverse *GRF9-IOE* specific primers. Genes tested by the chosen primer combinations are underlined. M, DNA size marker.
