# Supplementary material for: GROWTH-REGULATING FACTOR 9 negatively regulates arabidopsis leaf growth by controlling ORG3 and restricting cell proliferation in leaf primordia
Source: PLoS Genet. 2018 Jul 9;14(7):e1007484. doi: 10.1371/journal.pgen.1007484 (PMC6053248; doi:10.1371/journal.pgen.1007484)
Supplement: S10 Fig — (A) Mature petals of WT and org3-1 plants. (B) Petal size and (C) petal cell area. Data represent means ± SD from at least 32 petals (i.e., 4 petals from at least 8 plants). The asterisk indicates a significant difference from WT (Student's t-test; p < 0.05). Bar = 0.5 mm. (PDF) [file pgen.1007484.s014.pdf]

S10 Fig

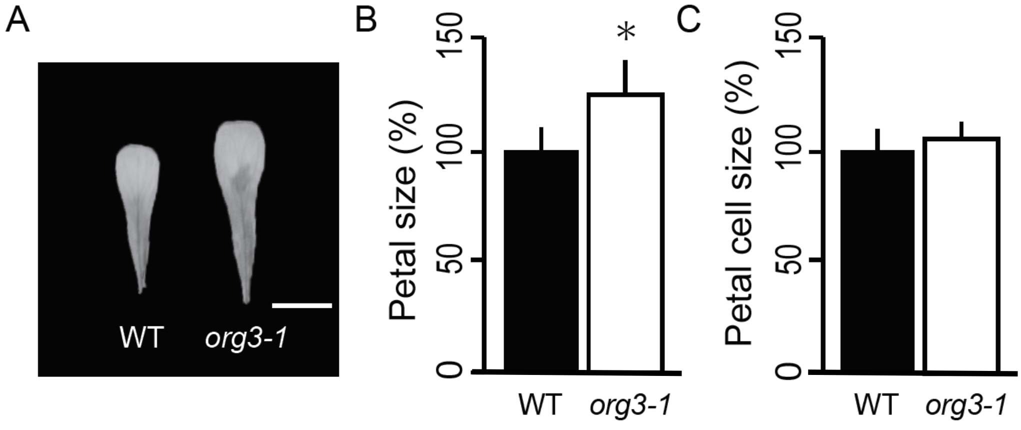

S10 Fig. Petal phenotype of the *org3-1* mutant. (A) Mature petals of WT and *org3-1* plants. (B) Petal size and (C) petal cell area. Data represent means ± SD from at least 32 petals (i.e., 4 petals from at least 8 plants). The asterisk indicates a significant difference from WT (Student´s *t*-test; $p < 0.05$). Bar = 0.5 mm.
